# Nature-Derived Epoxy Resin Monomers with Reduced Sensitizing Capacity—Isosorbide-Based Bis-Epoxides

**DOI:** 10.1021/acs.chemrestox.2c00347

**Published:** 2023-01-18

**Authors:** Isabella Karlsson, David J. Ponting, Miguel A. Ortega, Ida B. Niklasson, Lorena Ndreu, E. Johanna L. Stéen, Tina Seifert, Kristina Luthman, Ann-Therese Karlberg

**Affiliations:** † Department of Environmental Science, Exposure and Effect, Stockholm University, SE-106 91Stockholm, Sweden; ‡ Department of Chemistry and Molecular Biology, Dermatochemistry and Skin Allergy, University of Gothenburg, SE-412 96Gothenburg, Sweden; § Department of Chemistry and Molecular Biology, Medicinal Chemistry, University of Gothenburg, SE-412 96Gothenburg, Sweden

## Abstract

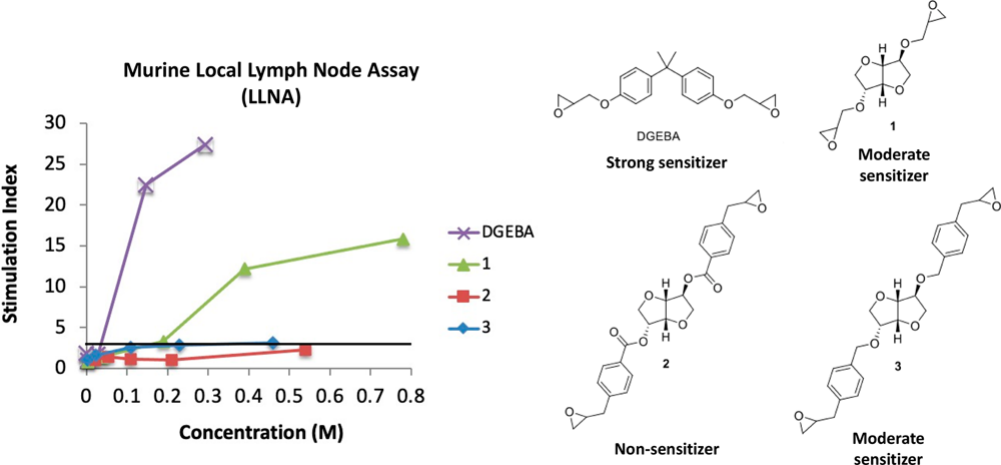

Epoxy resin systems (ERSs) are a class of thermosetting resins that become thermostable and insoluble polymers upon curing. They are widely used as components of protective surfaces, adhesives, and paints and in the manufacturing of composites in the plastics industry. The diglycidyl ether of bisphenol A (DGEBA) is used in 75–90% of ERSs and is thus by far the most used epoxy resin monomer (ERM). Unfortunately, DGEBA is a strong skin sensitizer and it is one of the most common causes of occupational contact dermatitis. Furthermore, DGEBA is synthesized from bisphenol A (BPA), which is a petroleum-derived chemical with endocrine-disruptive properties. In this work, we have used isosorbide, a renewable and nontoxic sugar-based material, as an alternative to BPA in the design of ERMs. Three different bis-epoxide isosorbide derivatives were synthesized: the diglycidyl ether of isosorbide (**1**) and two novel isosorbide-based bis-epoxides containing either a benzoic ester (**2**) or a benzyl ether linkage (**3**). Assessment of the in vivo sensitizing potency of the isosorbide bis-epoxides in the murine local lymph node assay (LLNA) showed that all three compounds were significantly less sensitizing than DGEBA, especially **2** which was nonsensitizing up to 25% w/v. The peptide reactivity showed the same order of reactivity as the LLNA, i.e., **2** being the least reactive, followed by **3** and then **1**, which displayed similar peptide reactivity as DGEBA. Skin permeation of **2** and **3** was compared to DGEBA using ex vivo pig skin and static Franz cells. The preliminary investigations of the technical properties of the polymers formed from **1**–**3** were promising. Although further investigations of the technical properties are needed, all isosorbide bis-epoxides have the potential to be less sensitizing renewable replacements of DGEBA, especially **2** that had the lowest sensitizing potency in vivo as well as the lowest peptide reactivity.

## Introduction

Epoxy resin systems (ERSs) are a class of thermosetting resins, i.e., soft solids or viscous liquids that become thermostable and insoluble polymers upon curing. The material formed from ERSs is strong, flexible, and lightweight; due to these attractive properties, ERSs have found a wide range of applications including adhesives, metal coatings, industrial flooring, and electrical insulators. Modern applications are relining of old pipes and wings of wind turbines. According to a report from the Zion Market Research, the worldwide market for epoxy resins accounted for USD 8.5 billion in 2020 and is expected to reach USD 14.7 billion by 2028.^1^

**Figure 1:**
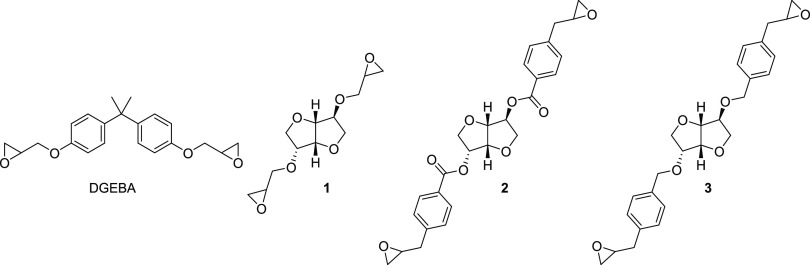
Structures of DGEBA and synthesized isosorbide-based bis-epoxides **1–3**.

The diglycidyl ether of bisphenol A (DGEBA) (Figure 1) is by far the most used epoxy resin monomer (ERM) in ERSs and constitutes 75–90% of all ERS polymers. Unfortunately, DGEBA is a strong skin sensitizer, which can give rise to allergic contact dermatitis (ACD). This makes DGEBA one of the most common causes of occupational contact dermatitis. After polymerization, the risk of sensitization decreases since the ERS polymers are too large to readily permeate the skin. However, small amounts of noncured ERMs, such as DGEBA, are always present in commercial ERS polymers and can cause adverse skin reactions even after curing. According to a German report, over a third of occupational diseases are skin diseases^2^ and the majority of these are irritant contact dermatitis or ACD.^3^ Contact allergy is caused by reactive chemicals, so-called haptens, which themselves are too small to be recognized by the immune system; however, by the modification of endogenous proteins in the skin, they are able to activate the immune system. It is a chronic disease for which there is no cure. The only way to avoid inflammation and eczema is to avoid skin contact with the compound responsible for the allergic reaction. DGEBA is included in the baseline series of about 30 common contact allergens that are used for the screening of ACD in dermatitis patients at dermatology clinics. The prevalence of positive eczematic reactions to DGEBA ranges from 0.9 to 2.3% when the compound is tested in consecutive dermatitis patients referred to the clinics.^4−8^ This places DGEBA on top of the most common contact allergens. In occupational settings, even higher prevalence of positive reactions to ERSs has been observed, sometimes as high as 10% or more.^3,9−14^ Although well-established and highly prioritized health and safety policies have been introduced in the wind turbine industry during the last 20 years, still about 10% of the workers involved in the manufacturing processes are sensitized to ERSs.^15,16^ Although the ERMs are considered the main sensitizers in ERSs, there are other components, such as diluents and hardeners, that also are reported to elicit allergic reactions.^17,18^ The skin-sensitizing potency of DGEBA has been assessed in the murine local lymph node assay (LLNA)^19^ as well as in the guinea pig maximization test,^20^ and in both of these in vivo models, DGEBA was classified as a strong skin sensitizer.^21^ Thus, it is of great importance to develop ERMs with lower skin sensitization potential without losing the technical polymerization properties. In our previous work, we studied the structure–activity relationship of ERMs^22−26^ and we could show that the terminal epoxides are the main reason for the sensitizing potency of DGEBA.^25^ Unfortunately, the terminal epoxide is necessary for the polymerization capacity of the ERMs and can therefore not be removed. We have, however, found that there are other structural features that can be changed in order to reduce the skin-sensitizing potency without significantly affecting the polymerization capacity.^24^ For instance, analogues in which the diglycidyl ether oxygen atom was removed, the aromaticity was removed, or the chain length was extended displayed reduced skin-sensitizing capacity in the LLNA without significantly impacting the thermal stability of the polymers formed with triethylenetetramine (TETA).^24^ Another concern regarding DGEBA is that it is synthesized from bisphenol A (BPA), a fossil fuel-derived chemical, which has been shown to have hormone-like properties.^27^

In the present project, we continue our work to develop ERMs with reduced sensitizing potency without compromising the polymerization properties. In addition, we wanted to find a more environmentally sustainable starting material compared to petroleum-derived BPA. Our choice for the ERM scaffold fell on isosorbide, which is a renewable sugar-derived compound that is used in the production of biobased polymers and has also found medical use as a diuretic.^28^ Isosorbide-based biomaterials have the advantage of being rigid and nontoxic; thus, we hypothesized that isosorbide-based ERMs would also possess these attractive properties. Three different bis-epoxides, based on the isosorbide scaffold, were designed and synthesized (Figure 1). The sensitizing potencies of these compounds were evaluated in vivo using the LLNA, in chemico toward the peptide AcPHCKRM, and in silico using density functional theory (DFT) calculations. Skin permeation was assessed in Franz diffusion cell experiments. In addition, preliminary investigations of the technical properties of the isosorbide bis-epoxides were performed by polymerization with TETA, followed by thermogravimetric analysis (TGA) of the formed polymers.

## Experimental Procedures

### Caution

This study involves skin-sensitizing compounds which should be handled with particular care.

### Instrumentation and Mode of Analysis

^1^H and ^13^C NMR spectroscopy was performed on a Varian 400 MR spectrometer at 400 MHz and 100 MHz, respectively, using CDCl_3_ (residual CHCl_3_ δ 7.26 and δ 77.0 as internal standards). All NMR experiments were performed at ambient temperature. Electron-ionization mass spectral analysis (70 eV) was performed on a Hewlett-Packard 5973 mass spectrometer connected to a gas chromatograph (Hewlett-Packard 6890). The GC was equipped with a splitless capillary inlet and an HP-5MS fused silica capillary column (30 m × 0.25 mm i.d., particle size, 0.25 μm, Agilent Technologies, Palo Alto, CA, USA). Helium was used as the carrier gas, and the flow rate was 1.1 mL/min. The inlet temperature was set at 250 °C. The temperature program started at 35 °C for 1 min, increased by 10 °C/min, and ended at 250 °C for 5 min. For mass spectral analysis, the mass spectrometer was used in the scan mode detecting ions with *m*/*z* values ranging from 50 to 1500.

Liquid chromatography–mass spectroscopy (LC–MS) analyses of peptide depletion experiments were performed using electrospray ionization (ESI) on a Hewlett-Packard 1100 HPLC/MS (Agilent Technologies, Waldbronn, Germany). The system included a vacuum degasser, a binary pump, an autoinjector, a column thermostat, a diode array detector, and a single quadrupole mass spectrometer. The high-performance liquid chromatography (HPLC) system was equipped with a HyPURITY C18 column (150 × 3 mm i.d., particle size 3 μm, Thermo Hypersil-Keystone, Thermo Electron Corp., Bellafonte, PA, USA). The mobile phase consisted of 0.005% pentafluoropropanoic acid, 0.1% acetic acid, and 5% acetonitrile in water (solvent A) and 0.005% pentafluoropropanoic acid, 0.1% acetic acid, and 50% water in acetonitrile (solvent B). Aliquots of 3 μL were injected onto the column and eluted with a flow rate of 0.40 mL/min and the column temperature was set to 40 °C. The gradient conditions used were as follows: 0–8% B (0–3 min), 8–10% B (3–15 min), 10–100% B (15–18 min), 100% B (18–30 min), 100–0% B (30–30.1 min), 0% B (30.1–40 min). The ESI interface was used in positive ionization mode with the following spray chamber settings: nebulizer pressure, 35 psi; capillary voltage, 3000 V; drying gas temperature, 350 °C; and drying gas flow rate, 12 L/min. The fragmentor voltage was set to 120 V. The mass spectrometer was used in scan mode detecting molecular ions with *m*/*z* values ranging from 50 to 1800.

For the permeability experiments, ultra-performance liquid chromatography-electrospray ionization tandem mass spectroscopy (UPLC)-ESI-MS/MS analysis was carried out using a Xevo TQ-S micro triple quadrupole mass spectrometer equipped with an electrospray interface and coupled to an Acquity UPLC H-Class Plus system from Waters. Separation was achieved on an ACE Excel C18-PFP column (75 × 2.1 mm, 1.7 μm) from Advanced Chromatography Technologies Ltd. (Aberdeen, Scotland). Mobile phase A consisted of 0.1% formic acid and 5% acetonitrile in water, whereas mobile phase B consisted of 0.1% formic acid and 5% water in acetonitrile. The gradient program used was as follows: 20% B (0–2 min), 20–80% B (2–9 min), 80–95% B (9–9.1 min), 95% B (9.1–12 min), 95–20% B (12–12.1 min), 20% B (12.1–15 min). The flow rate was set to 0.3 mL/min and the injection volume was 2 μL. Analysis was performed in positive ionization mode using a Multiple Reaction Monitoring (MRM) method. The optimal parameters were capillary voltage 1.5 (kV), desolvation temperature 650 °C, desolvation gas 850 L/h, and cone 100 L/h. Table S2 shows the specific compound information used in the method.

High-resolution mass spectrometry (HRMS) analyses were performed using an Orbitrap Q Exactive HF mass spectrometer (Thermo Fisher Scientific, MA, USA) interfaced to a Dionex UltiMate 3000 UHPLC system (Thermo Fisher Scientific, MA, USA). Mobile phase A consisted of 0.1% formic acid and 5% acetonitrile in water, whereas mobile phase B consisted of 0.1% formic acid and 5% water in acetonitrile. Chromatographic separation was performed using an Aquity UPLC HSS C18 column (2.1 × 100 mm, 1.8 μm) from Waters and a flow rate of 0.30 mL/min. The gradient started at 20% B for 0.5 min, followed by an increase to 100% B in 8 min, and finally, the solvent composition was held at 100% B for 3.5 min before re-equilibration for 2.5 min. The MS was used in full scan mode detecting positive ions ranging from *m/z* 50 to 750. The resolution was set to 120,000, the AGC target to 3 × 10,^6^ and the maximum IT to 200 ms. The tune parameters were as follows: spray voltage, 4 kV; capillary temperature, 275 °C; sheath gas, 20 arbitrary units (au); auxiliary gas, 10 au; S-Lens RF level, 60%; probe heater temperature, 240 °C.

### Chemistry

Ac-Pro-His-Cys-Lys-Arg-Met-OH (AcPHCKRM, 98%) was obtained from Peptide 2.0 Inc. (Chantilly, Virginia, USA). Acetone p.a. was purchased from Merck (Darmstadt, Germany) and olive oil from Apoteket AB (Gothenburg, Sweden). [Methyl-^3^H]thymidine (2.0 Ci/mmol) was obtained from Perkin-Elmer Biosciences (Waltham MA, USA). All starting materials and reagents were obtained from commercial suppliers and were used without prior purification. Tetrahydrofuran (THF) and CH_2_Cl_2_ used in reactions with anhydrous conditions were distilled from Na and CaH_2_, respectively. All reactions were monitored by thin-layer chromatography (TLC) on silica-plated aluminum sheets (Silica gel 60 F254, E. Merck). Spots were detected by UV light (254 or 365 nm), or developed with heating, or anisaldehyde or potassium permanganate staining. All reactions were carried out using magnetic stirring under an ambient atmosphere if not otherwise noted. Microwave reactions were carried out using a Biotage Initiator Sixty in 10–20 mL capped microwave vials with fixed hold time, normal or high absorption, and 10–30 s pre-stirring. Purification by flash column chromatography was performed using Merck silica gel Geduran Si 60 (0.063–0.200 mm) or by using an automatic Biotage SP4 Flash^+^ instrument with pre-packed columns (surface area 500 m^2^/g, porosity 60 Å, particle size 40–63 μm).

#### (3*R*,3a*R*,6*S*,6a*R*)-3,6-*O*-Bis(3,4-epoxypropyl)-1,4:3,6-dianhydro-d-glucitol (**1**) (Scheme 1)

##### (3*R*,3a*R*,6*S*,6a*R*)-3,6-*O*-Diallyl-1,4:3,6-dianhydro-d-glucitol (**1a**)

A procedure was adapted from the literature^29^ as follows: isosorbide (1 g, 6.84 mmol) was reacted with allyl bromide (2.07 mL, 23.95 mmol) at 65 °C under stirring for 1 h. Then, NaOH (0.96 g, 23.95 mmol) dissolved in water (10 mL) was added dropwise and the reaction mixture was heated at 65 °C for 90 min. The reaction was monitored by TLC (CH_2_Cl_2_/MeOH 95:5). Additional allyl bromide (1.2 mL, 13.68 mmol) was added and the reaction mixture was stirred at 65 °C for 4 h. Another portion of allyl bromide (1.2 mL, 13.68 mmol) was added and the mixture was stirred at room temperature overnight. The reaction mixture was poured into ice/water and was extracted with CH_2_Cl_2_. The organic layer was washed with diluted HCl, then with distilled water, dried (Na_2_SO_4_), filtered, and concentrated to give an oil which was purified by column chromatography (CH_2_Cl_2_/MeOH up to 1%) to afford **1a** (0.51 g, 33%). ^1^H NMR δ 6.00–5.84 (m, 2H), 5.32 (dq, *J* = 7.1, 1.6 Hz, 1H), 5.27 (dq, *J* = 7.1, 1.6 Hz, 1H), 5.20 (dddt, *J* = 10.4, 5.3, 1.3 Hz, 2H), 4.63 (t, *J* = 4.4 Hz, 1H), 4.52 (dd, *J* = 4.3, 1.1 Hz, 1H), 4.24–4.18 (m, 1H), 4.08–4.00 (m, 5H), 3.99–3.91 (m, 3H), 3.60 (ddd, *J* = 8.5, 8.1, 0.5 Hz, 1H).

##### (3*R*,3a*R*,6*S*,6a*R*)-3,6-*O*-Bis(3,4-epoxypropyl)-1,4:3,6-dianhydro-d-glucitol (**1**)

To **1a** (0.507 g, 2.24 mmol) dissolved in CHCl_3_ (15 mL), 3-chloroperbenzoic acid (*m*-CPBA, 1.55 g, 8.96 mmol) was added and the reaction mixture was stirred at room temperature overnight. The reaction was monitored by TLC (CH_2_Cl_2_/MeOH 95:5). The reaction mixture was filtered to remove a white solid, and to the filtrate, more *m*-CPBA (1.55 g, 8.96 mmol) was added. When no starting material was observed in TLC, 10% aq. NaOH was added and the mixture was stirred for 10 min. CH_2_Cl_2_ was added and the organic layer was separated, washed with brine, dried (Na_2_SO_4_), filtered, and concentrated to afford a crude product, which was purified by column chromatography to give **1** (0.50 g, 87%). The NMR data were in accordance with that reported in the literature.^29−31^^1^H NMR δ 4.70–4.62 (m, 1H), 4.52 (ddt, *J* = 11.4, 4.4, 0.9 Hz, 1H), 4.16–3.90 (m, 6H), 3.87–3.78 (m, 2H), 3.66–3.57 (m, 2H), 3.48–3.35 (m, 2H), 3.22–3.11 (m, 2H), 2.80 (ddddt, *J* = 4.9, 4.0, 3.3, 2.4, 0.8 Hz, 2H), 2.65–2.57 (m, 2H). ^13^C NMR δ 86.3, 86.22, 86.21, 86.15, 84.92, 84.89, 84.88, 84.8, 80.74, 80.71, 80.44, 80.40, 80.2, 73.45, 73.43, 73.25, 73.21, 71.8, 71.12, 71.11, 70.4, 70.31, 70.27, 70.1, 69.8, 50.87, 50.85, 50.7, 50.6, 44.3, 44.2, 44.12, 44.11.

#### (3*R*,3a*R*,6*S*,6a*R*)-3,6-*O*-Bis[4-(3,4-epoxypropyl)benzoyl]-1,4:3,6-dianhydro-d-glucitol (**2**) (Scheme 2)

##### (3*R*,3a*R*,6*S*,6a*R*)-3,6-*O*-Bis[4-iodobenzoyl]-1,4:3,6-dianhydro-d-glucitol (**2a**)

4-Iodobenzoic acid (6.45 g, 26.0 mmol) was dissolved in dry THF (120 mL) and 1,1′-carbonyldiimidazole (CDI) (4.31 g, 26.0 mmol) was added at room temperature. The mixture was heated to 60 °C for 1 h and isosorbide (1.70 g, 11.6 mmol) was added. The mixture was continued to stir at 60 °C for 22 h. The reaction mixture was allowed to cool to room temperature and was concentrated. The off-white crude product was purified by automated flash column chromatography using CH_2_Cl_2_ as the eluent to give **2a** (5.96 g, 86%) as a white solid. ^1^H NMR δ 7.84–7.74 (m, 6H), 7.72–7.67 (m, 2H), 5.45 (td, *J* = 2.3, 1.1 Hz, 1H), 5.39 (td, *J* = 5.6, 4.9 Hz, 1H), 5.03 (t, *J* = 5.1 Hz, 1H), 4.65 (dd, *J* = 4.8, 0.9 Hz, 1H), 4.11–3.97 (m, 4H). ^13^C NMR δ 165.6, 165.3, 138.02, 137.99, 131.3, 131.2, 129.03, 128.99, 101.5, 101.4, 86.2, 81.2, 76.8, 74.8, 73.5, 70.9.

##### (3*R*,3a*R*,6*S*,6a*R*)-3,6-*O*-Bis[4-allylbenzoyl]-1,4:3,6-dianhydro-d-glucitol (**2b**)

A mixture of **2a** (0.523 g, 0.863 mmol), CsF (0.508 g, 3.348 mmol), and Pd(PPh_3_)_4_ (0.1 g, 0.086 mmol) in THF (15 mL) was stirred for 30 min at room temperature. Allylboronic acid pinacol ester (0.583 mL, 3.107 mmol) in THF (5 mL) was added and the reaction mixture was heated under reflux overnight. The day after, a thick yellow suspension had formed. The reaction was monitored by TLC (pentane/EtOAc 80:20). After 24 h, the mixture was allowed to reach room temperature, and CH_2_Cl_2_ and water were added. The phases were separated. The aqueous phase was extracted with CH_2_Cl_2_ (2x) and the combined organic phases were washed with water and brine, dried (Na_2_SO_4_), filtered, and concentrated to give a sticky yellow crude product, which was purified by column chromatography (isocratic pentane/EtOAc 80:20) to afford **2b** (0.331 g, 88%) as an ochre solid. ^1^H NMR δ 8.04–7.98 (m, 2H), 7.98–7.91 (m, 2H), 7.31–7.23 (m, 4H), 5.95 (ddtd, *J* = 16.9, 10.2, 6.7, 5.6 Hz, 2H), 5.50–5.38 (m, 2H), 5.16–5.02 (m, 5H), 4.68 (dt, *J* = 4.7, 0.8 Hz, 1H), 4.16–4.00 (m, 4H), 3.50–3.40 (m, 4H). ^13^C NMR δ 166.1, 165.7, 146.2, 146.1, 136.44, 136.38, 130.13, 130.09, 128.87, 128.85, 127.55, 127.53, 116.83, 116.80, 86.4, 81.3, 78.5, 74.5, 73.7, 70.9, 40.31, 40.29.

##### (3*R*,3a*R*,6*S*,6a*R*)-3,6-*O*-Bis[4-(3,4-epoxypropyl)benzoyl]-1,4:3,6-dianhydro-d-glucitol (**2**)

A solution of **2b** (200 mg, 0.46 mmol) in dry CH_2_Cl_2_ (6 mL) in an oven-dried flask was purged with N_2_ for 10 min and cooled to 0 °C in an ice bath. *m*-CPBA (412 mg, 1.84 mmol) was added in one portion and the mixture was continued to stir for 21 h while the cooling bath was slowly warming. The mixture was diluted with CH_2_Cl_2_ and 1 M NaOH (aq.) was added. The phases were separated and the aqueous phase was extracted with CH_2_Cl_2_ (×3). The combined organic phases were washed with brine, dried (MgSO_4_), filtered and concentrated to give the crude product as colorless oil. The crude product was purified by automated flash column chromatography using EtOAc/toluene to give the desired product (140 mg, 65%). ^1^H NMR δ 8.06–8.00 (m, 2H), 7.99–7.92 (m, 2H), 7.39–7.28 (m, 4H), 5.50–5.46 (m, 1H), 5.41 (q, *J* = 5.5 Hz, 1H), 5.05 (t, *J* = 5.1 Hz, 1H), 4.67 (d, *J* = 4.7 Hz, 1H), 4.18–3.96 (m, 4H), 3.22–3.09 (m, 2H), 3.00–2.85 (m, 4H), 2.80 (dt, *J* = 4.9, 3.6 Hz, 2H), 2.57–2.49 (m, 2H). ^13^C NMR δ 165.9, 165.5, 143.3, 143.3, 143.1, 130.13, 130.10, 129.27, 129.25, 128.10, 128.07, 86.3, 81.3, 78.54, 74.6, 73.6, 70.9, 52.01, 51.97, 51.96, 46.82, 46.79, 38.80, 38.78, 38.76. HRMS (Orbitrap ESI) calculated for C_26_H_27_O_8_^+^ 467.1700, found 467.1707 (1.5 ppm).

#### (3*R*,3a*R*,6*S*,6a*R*)-3,6-*O*-Bis[4-(3,4-epoxypropyl)benzyl]-1,4:3,6-dianhydro-d-glucitol (**3**) (Scheme 3)

##### (3*R*,3a*R*,6*S*,6a*R*)-3,6-*O*-Bis(4-iodobenzyl)-1,4:3,6-dianhydro-d-glucitol (**3a**)

A procedure was adapted from the literature^32^ as follows: Isosorbide (312 mg, 2.14 mmol), 4-iodobenzyl bromide (1.90 g, 6.4 mmol), tetrabutylammonium bromide (72.0 mg, 0.22 mmol), and KOH (353 mg, 6.41 mmol) were suspended in *o*-xylene (11 mL) and heated in a microwave reactor at 125 °C for 1 h. The mixture was diluted with CH_2_Cl_2_ and the salts were filtered off. The filtrate was concentrated under reduced pressure. The crude product was purified by flash column chromatography using EtOAc in pentane (3,7) as the eluent to afford **3a** (959 mg, 78%) as a white solid. ^1^H NMR δ 7.66 (dd, *J* = 8.3, 1.6 Hz, 4H), 7.08 (dd, *J* = 17.1, 8.2 Hz, 4H), 4.76–4.61 (m, 2H), 4.59–4.39 (m, 4H), 4.07–4.02 (m, 2H), 4.02–3.95 (m, 2H), 3.86 (dd, *J* = 8.8, 6.6 Hz, 1H), 3.62 (dd, *J* = 8.8, 7.6 Hz, 1H). ^13^C NMR δ 137.6, 137.6, 137.5, 137.5, 137.4, 137.4, 129.8, 129.6, 93.5, 93.5, 93.43, 93.42, 86.4, 84.0, 80.4, 79.4, 73.4, 71.8, 70.9, 70.2.

##### (3*R*,3a*R*,6*S*,6a*R*)-3,6-*O*-Bis(4-allylbenzyl)-1,4:3,6-dianhydro-d-glucitol (**3b**)

A procedure adapted from the literature^33^ was used as follows: A solution of **3a** (516 mg, 0.89 mmol), Pd(PPh_3_)_4_ (206 mg, 0.18 mmol), and CsF (542 mg, 3.57 mmol) in dry THF (15 mL) was stirred at room temperature for 1 h under a N_2_ atmosphere. Allylboronic acid pinacol ester (0.7 mL, 3.7 mmol) was added and the mixture was heated to reflux for 17 h under a N_2_ atmosphere. CH_2_Cl_2_ and water were added to the mixture. The phases were separated and the aqueous phase was extracted with CH_2_Cl_2_. The combined organic phases were washed with brine, dried (MgSO_4_), filtered, and concentrated under reduced pressure. The crude product was purified by flash column chromatography using EtOAc in toluene (0 → 20%) as the eluent to afford **3b** (276 mg, 76%) as a yellow oil. ^1^H NMR δ 7.38–7.22 (m, 4H), 7.21–7.12 (m, 4H), 6.09–5.88 (m, 2H), 5.29–4.90 (m, 4H), 4.75 (d, *J* = 11.8 Hz, 1H), 4.67 (t, *J* = 4.4 Hz, 1H), 4.60–4.48 (m, 4H), 4.14–3.93 (m, 4H), 3.85 (dd, *J* = 8.7, 6.7 Hz, 1H), 3.61 (t, *J* = 8.3 Hz, 1H), 3.39 (dd, *J* = 6.7, 1.6 Hz, 4H). ^13^C NMR δ 139.92, 139.87, 137.44, 137.42, 135.6, 135.5, 128.83, 128.81, 128.3, 128.1, 116.0, 86.5, 83.9, 80.4, 79.1, 73.5, 72.3, 71.5, 70.1, 40.1, 40.0.

##### (3*R*,3a*R*,6*S*,6a*R*)-3,6-*O*-Bis[4-(3,4-epoxypropyl)benzyl]-1,4:3,6-dianhydro-d-glucitol (**3**)

*m*-CPBA (308 mg, 1.79 mmol) was added in one portion to a solution of **3b** (171 mg, 0.42 mmol) in dry CH_2_Cl_2_ (11 mL) at 0 °C. The mixture was stirred under a N_2_ atmosphere overnight, while slowly reaching room temperature. The reaction was treated with 1 M NaOH and diluted with CH_2_Cl_2_. The phases were separated and the aqueous phase was extracted with CH_2_Cl_2_. The combined organic phases were washed with water and brine, dried (MgSO_4_), filtered, and concentrated under reduced pressure. The crude product was purified by flash column chromatography with EtOAc in pentane (0 → 2:3) as the eluent. In cases where mono-epoxide was detected, purification by flash column chromatography was performed on spherical silica gel (Biotage SNAP Ultra 100 g, HP-Sphere, particle size 25 μm). **3** was afforded (153 mg, 83%) as a yellow oil. ^1^H NMR δ 7.35–7.20 (m, 8H), 4.74 (d, *J* = 11.8 Hz, 1H), 4.67 (t, *J* = 4.5 Hz, 1H), 4.59–4.48 (m, 4H), 4.13–3.93 (m, 4H), 3.86 (dd, *J* = 8.7, 6.7 Hz, 1H), 3.61 (t, *J* = 8.3 Hz, 1H), 3.13 (dp, *J* = 5.7, 3.2 Hz, 2H), 2.97–2.68 (m, 6H), 2.54 (dd, *J* = 5.0, 2.6 Hz, 2H). ^13^C NMR δ 136.98, 136.94, 136.3, 136.1, 129.24, 129.23, 128.4, 128.3, 128.1, 86.5, 83.9, 80.4, 79.2, 77.5, 77.4, 76.8, 73.5, 72.3, 71.4, 70.1, 52.5, 46.9, 38.6. HRMS (Orbitrap ESI) calculated for C_21_H_36_O_6_^+^ 439.2115, found 439.2121 (1.3 ppm).

### Experimental Animals

Female CBA/Ca mice, 7 to 9 weeks of age, were purchased from NOVA SCB Charles River, Germany. The mice were housed in HEPA-filtered air flow cages and kept on standard laboratory diet and water ad lib. The regional ethics committee, Jordbruksverket, approved the protocol and the procedure was performed in accordance with the guidelines.

### Skin-Sensitizing Potency of Isosorbide Bis-Epoxides 1–3 in Mice

A previously published protocol^24^ of the LLNA^34^ was used to assess the sensitization potential of the isosorbide bis-epoxides **1–3**. The purity of the assessed isosorbide bis-epoxides was >99% (GC/MS or LC/MS) before testing of sensitization potential. The isosorbide bis-epoxides were tested in five different concentrations; **1**: 3.9 mM (0.10% w/v), 39 mM (1.0% w/v), 190 mM (5.0% w/v), 390 mM (10% w/v), and 770 mM (20% w/v); **2**: 21 mM (1.0% w/v), 54 mM (2.5% w/v), 110 mM (5.0% w/v), 210 mM (10% w/v), and 540 mM (25% w/v); and **3**: 2.3 mM (0.10% w/v), 23 mM (1.0% w/v), 110 mM (5.0% w/v), 230 mM (10% w/v), and 460 mM (20% w/v). A detailed description is given in Supporting Information.

### Peptide Depletion with AcPHCKRM

All solvents were degassed with argon prior to use. Solutions of **1–3** or **DGEBA** in dimethyl sulfoxide (DMSO) (40 mM, 75 μL) together with potassium phosphate buffer (100 mM, pH 7.4) (150 μL) were added to vials purged with argon containing AcPHCKRM in DMSO (4 mM, 75 μL). Accordingly, the final concentrations of test compounds and the model peptide in the reaction mixture were 10 mM and 1 mM, respectively. The reaction was kept at room temperature and was monitored with HPLC/ESI-MS every 40 min for 24 h, with the amount of the remaining peptide determined by ion extraction (*m/z* = 407.2, [M + 2H]^2+^).

### Assessment of the Skin Permeability of Isosorbide Bis-Epoxides **2** and **3**

The Franz diffusion cell experiment was based on the OECD Test Guidelines.^35^ Static Franz diffusion cells equipped with pig ex vivo epidermis were used to assess the permeability of isosorbide bis-epoxides **2** and **3**. Freshly cut pig ears were received frozen and stored at −20 °C until used for permeability assessment. One day before the experiment, the ears were transferred at +4 °C and allowed to thaw. The ears were then washed, shaved, and carefully cut with a Watson Dermatome skin graft knife to obtain 0.1 mm pieces of the epidermis layer. The integrity of the skin (transparent and intact) was determined by ocular inspection. Thereafter, the epidermis layer was cut and positioned between the donor and receptor compartment of each Franz cell. The receptor compartments were filled with 5 mL of ethanol/PBS (1:1) and equipped with a small magnetic stir bar to ensure mixing while maintaining a constant temperature of the circulating water at 37 °C. To equilibrate the skin, the upper chamber was filled with 0.5 mL of ethanol/PBS (1:1) which was later removed and replaced with the donor solution (50 μL) containing either **2** or **3** (0.1 mg/mL) or DGEBA (0.1 mg/mL) used as a positive control. A procedural blank containing only 50 μL of ethanol/PBS (1:1) in the donor compartment was also included in the experimental setup. Samples of 100 μL were collected after 0–2 min, 1, 2, 3, 4, 5, and 6 h. At each timepoint, a sample aliquot was removed and 100 μL of ethanol/PBS (1:1) was added to the receptor compartment to keep the volume constant. After 6 h, the skin from each Franz cell was extracted overnight in 5 mL of ethanol/PBS (1,1). Triplicate experiments were performed for each compound. The obtained samples were analyzed with UPLC-ESI-MS/MS (see Figure S2 for parameters) and quantification was based on external calibration curves. The limit of quantification (LOQ) was determined by the analysis of the analyte at low concentration and was calculated as 10 times the signal-to-noise ratio.

### Computational Techniques

The reactivity of cysteine residues toward compounds under investigation was modeled as reactivity toward methanethiolate. Reactivity calculations were carried out at the B3LYP-D3/6-31+G**^36−41^ level of theory in Jaguar (Schrodinger LLC, N. Y. (2009) Jaguar, version 7.6). Implicit solvation was modeled using the Poisson–Boltzmann finite system; however, in most cases, at least one explicit water molecule was also included since these have been shown to be implicated in the most energetically favored transition states (c.f. Ponting et al. 2019 Figure 6).^26^ Transition states were deduced initially using the linear synchronous transit method; however, those that proved less easy to find were discovered using a variety of techniques including temporarily constraining the locations of reactive species and variation of the initial guess. Initial preparation of structures was carried out using MacroModel (Schrodinger LLC, N. Y. (2009) Jaguar, version 9.7) and Maestro (Schrodinger LLC, N. Y. (2009) Jaguar, version 9.0). Calculations were performed on a mixture of the C3SE cluster (SNIC facility located at the Chalmers University of Technology, Gothenburg) and standalone workstations running CentOS 6.6. For isosorbide bis-epoxides **2** and **3**, the structures were truncated by reducing the isosorbide core to a methyl group for computational efficiency.

### Polymerization Procedure

The polymerization reaction between the ERMs DGEBA and compounds **1–3** with TETA was performed as previously described.^24^ Detailed information on the polymerization procedure and the thermogravimetric analysis (TGA) is given in the Supporting Information.

## Results

### Design and Synthesis

**Scheme 1:**
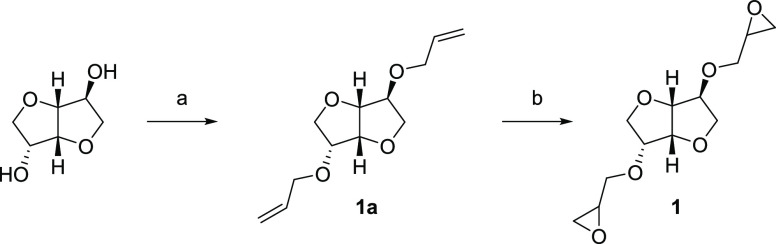
Synthesis of Compound 1^*a*^ Reagents and conditions: (a) Allyl bromide, NaOH, 65 °C → room temp., overnight; (b) *m*-CPBA, CHCl_3_, room temp., overnight.

**Scheme 2:**
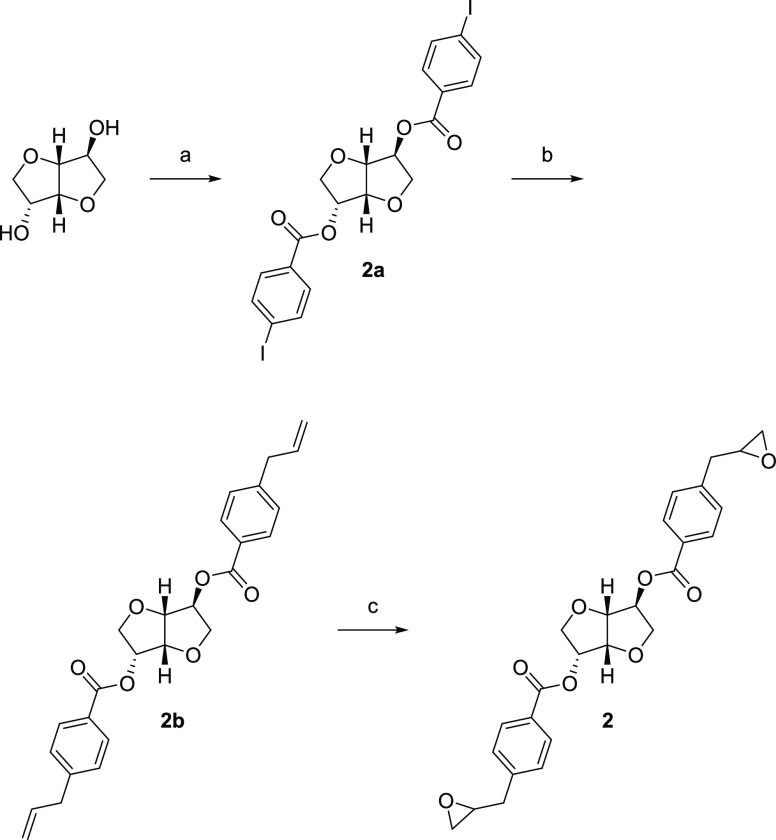
Synthesis of Compound 2^*a*^ Reagents and conditions: (a) 4-Iodobenzoic acid, CDI, THF, 60 °C, 23 h; (b) (i) Pd(PPh_3_)_4_, CsF, THF, room temp., 30 min. (ii) Allylboronic acid pinacol ester, THF, reflux, overnight; (c) *m*-CPBA, CH_2_Cl_2_, 0 °C → room temp., 21 h.

**Scheme 3:**
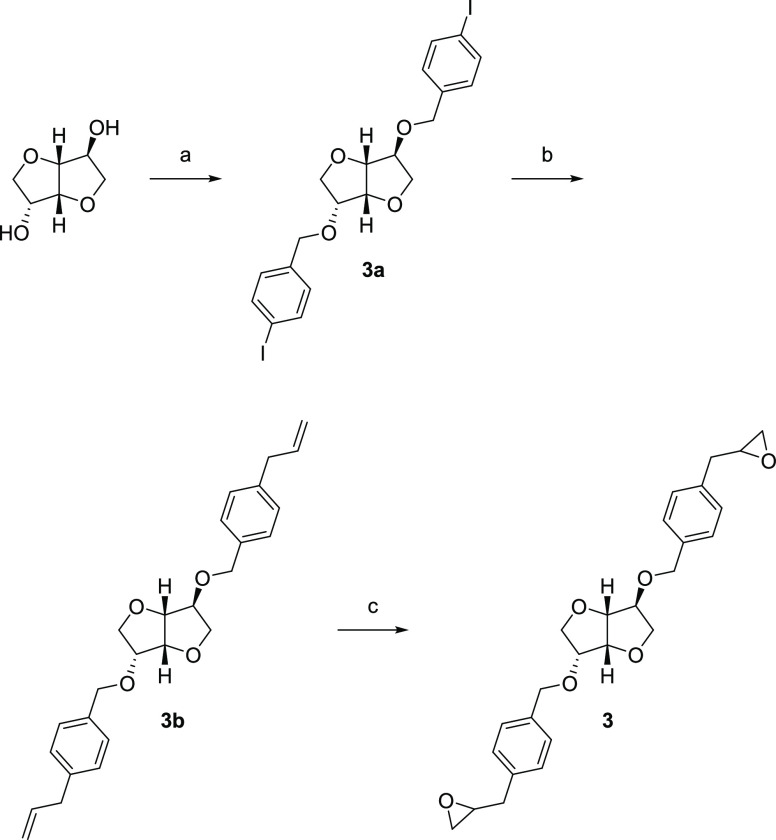
Synthesis of Compound 3^*a*^ Reagents and conditions: (a) 4-Iodobenzyl bromide, TBAB, KOH, *o*-xylene, 125 °C, MW, 1 h; (b) (i) Pd(PPh_3_)_4_, CsF, THF, room temp., 1 h. (ii) Allylboronic acid pinacol ester, THF, reflux, 17 h; (c) *m*-CPBA, CH_2_Cl_2_, 0 °C → room temp., 24 h.

The diglycidyl ether of isosorbide (**1**) and two novel isosorbide-based bis-epoxides (**2** and **3**) were synthesized (Schemes 1^2^,3). Isosorbide bis-epoxide **2** contains a benzoic acid ester and isosorbide bis-epoxide **3** a benzyl ether linkage. The synthesis of **1** proceeded via a bis-allylation of isosorbide^29^ (allyl bromide, NaOH) followed by epoxidation with *m*-CPBA (Scheme 1). Bis-esterification of isosorbide using 4-iodobenzoic acid and CDI provided **2a** (Scheme 2), which was then allylated using a Suzuki coupling with allylboronic acid pinacol ester to obtain **2b**. A final epoxidation gave **2**. A similar procedure was used to synthesize **3**, starting with bis-benzylation of isosorbide (Scheme 3).

### Skin Sensitization Potency

**Figure 2:**
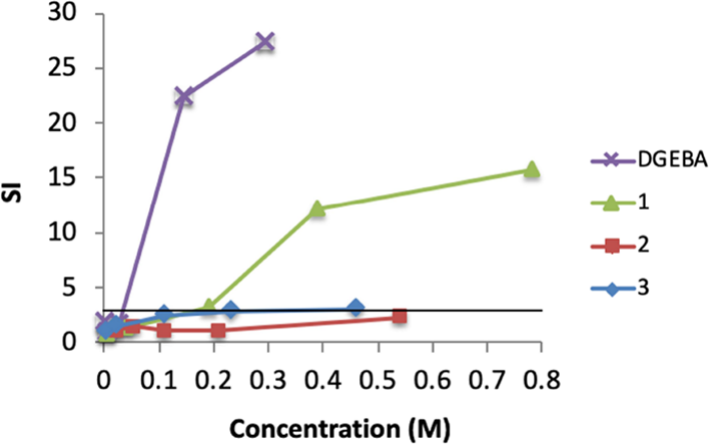
Results from the LLNA for bis-epoxides **1–3**. Dose–response curves for DGEBA (purple cross), **1** (green triangle), **2** (red square), and **3** (blue diamond). SI = stimulation index. EC3 values, DGEBA: 0.036 M; **1**: 0.17 M; **2**: nonsensitizing in concentrations up to 25% (w/v); **3**: 0.36 M.

The three isosorbide bis-epoxides were evaluated for sensitization potential in vivo with the murine LLNA (Figure 2). The stimulation index (SI) is defined as the ratio between the dpm/lymph node for the test group and the control group. Test materials that at one or more concentrations cause an SI greater than 3 are considered positive in the LLNA. The estimated concentration required to induce an SI of 3 (EC3) is used to determine the sensitizing potency of the test compound. The sensitizing potency was classified as follows: EC3 ≤ 0.2%, extreme; 0.2% < EC3 ≤ 2%, strong; EC3 > 2%, moderate.^21^ Isosorbide bis-epoxide **1** gave an EC3 value of 0.17 M (4.4%), **3** gave an EC3 value of 0.36 M (16%), and no EC3 value was obtained for **2** (for full results, see the Supporting Information, Table S1). This classifies both **1** and **3** as moderate skin sensitizers, whereas **2** was found to be nonsensitizing in concentrations up to 25% w/v (0.54 M).^21^

### Peptide Depletion with AcPHCKRM

**Figure 3:**
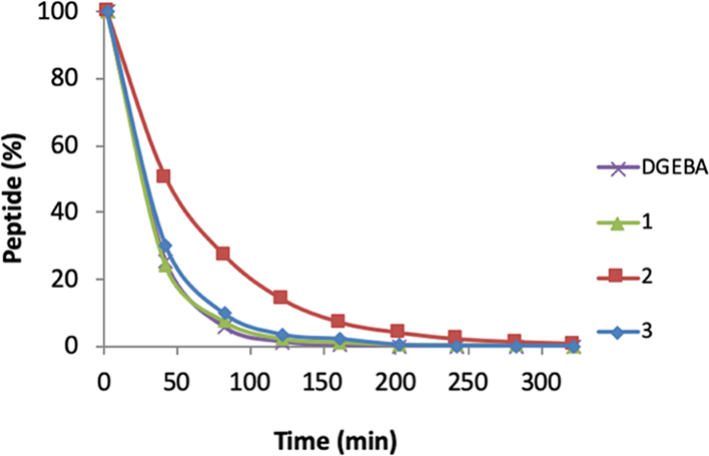
Depletion of the model peptide AcPHCKRM (1 mM) during 5 h in the presence of ten-fold excess (10 mM) of DGEBA (purple cross), **1** (green triangle), **2** (red square), and **3** (blue diamond) in DMSO/phosphate buffer pH 7.4 (1:1).

**1** showed the highest reactivity with the model peptide AcPHCKRM and followed the same depletion curve as DGEBA, **3** was slightly less reactive, whereas **2** was found to be the least reactive (Figure 3). The stability of the peptide AcPHCKRM, under the experimental conditions used, has previously been verified and no degradation was observed within 24 h.^42^ However, in the present study, some dimerization was observed in the absence of a hapten, but this slow reaction was out-competed by peptide modification. The stability of **1–3** was explored in a similar manner to exclude any risk of hydrolysis of the epoxides or ester, but no degradation was observed within 24 h under the conditions of the reactivity experiment.

### Skin Permeability

**Figure 4:**
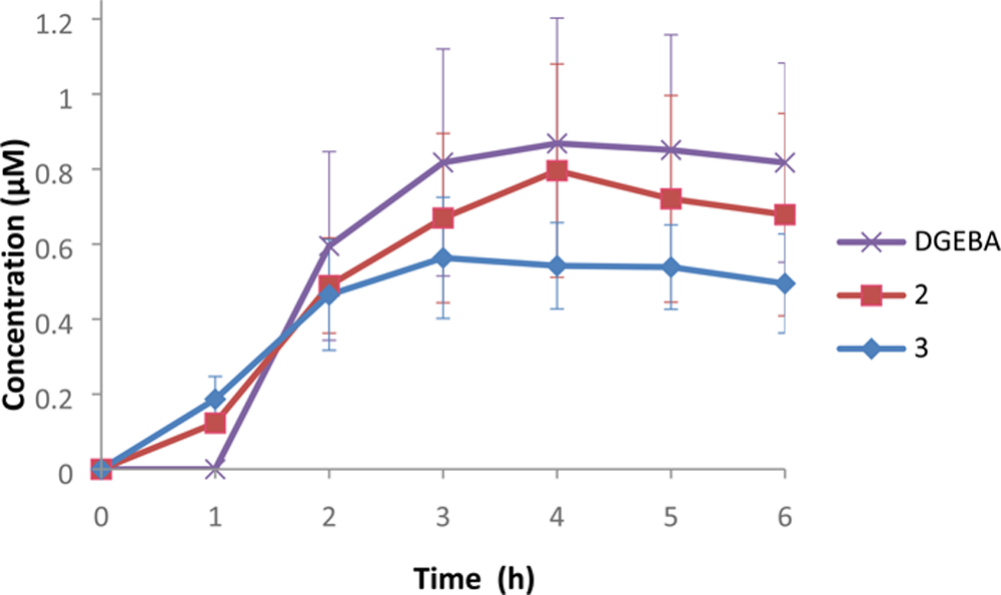
Results from the 6 h skin permeability experiment using static Franz cells fitted with ex vivo pig epidermis. The chart shows the concentration (μM) in the receptor fluid of DGEBA (purple cross), **2** (red square), and **3** (blue diamond).

Permeation of epidermis was observed for **2** and **3**, as well as for DGEBA, used as the positive control (Figure 4, Tables S3–S5). All compounds showed similar permeation rates at all tested time points with **3** exhibiting slightly lower permeability. However, due to the nature of the experiment and possible variations in the epidermal layers used in each replicate, the differences between the compounds were not significant. Among the three investigated compounds, DGEBA had the highest LOQ (160 pg. on column compared to 1 pg. for **2** and 11 pg. for **3)**, not allowing quantification at the one-hour time point. The recovery for each compound was calculated by summing the amount in the receptor compartment after 6 h with the amount after the overnight skin extraction. It varied from 48% for **2**, 35% for DGEBA, and 25% for **3** (Table 1).

**Table 1:** Recovery Expressed in w/w % for the Compounds Tested in the Permeability Experiment with Static Franz Cells and Ex Vivo Pig Epidermis

test compound	amount on/in skin (%)	amount in receptor fluid after 6 h (%)	recovery (%)
DGEBA	7.0 ± 0.4	27.8 ± 9.0	34.8 ± 9.4
**2**	16.1 ± 3.0	31.7 ± 12.6	47.7 ± 15.6
**3**	3.2 ± 2.6	21.7 ± 5.8	24.9 ± 8.4

### In Silico Reactivity

**Figure 5:**
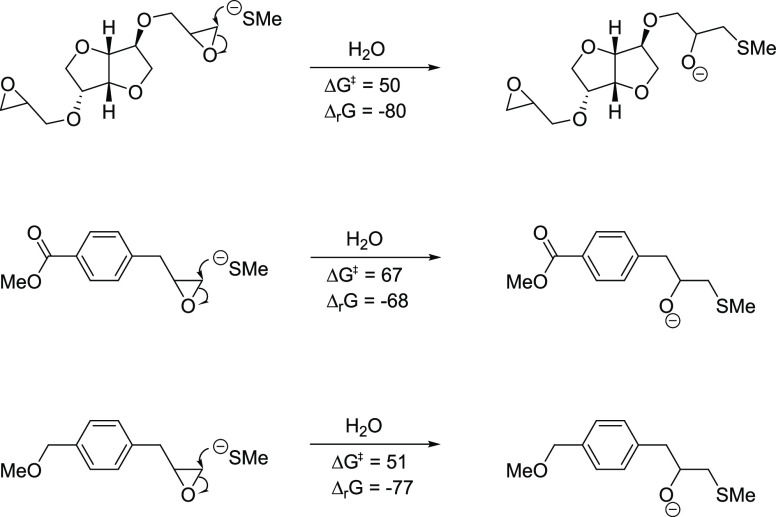
Reactivity parameters (Δ*G*^‡^, free energy of activation, and Δ_r_*G*, free energy change of reaction) for the attack of a model nucleophile on the epoxy groups of truncated structures of **1–3**.

Isosorbide bis-epoxide **2** is less reactive than **1** and **3** according to peptide reactivity experiments. The calculated reactivity of **1**, as compared to **2**, supports our earlier assessment that the absence of glycidyl ether oxygen reduces the reactivity.^24^ Also, the higher reactivity of **3** compared to **2** is supported by the results from the calculations reported here (Figure 5). As discussed in previous work,^26^ weaker interaction with a more electron-poor aromatic ring (as in **2**) leads to a higher activation energy for the epoxide ring opening and thus a lower reactivity; the more electronically neutral ring in **3** has comparable activation energy to the oxygen-interacting epoxide in **1**.

### Properties of the ERM Polymers

**Figure 6:**
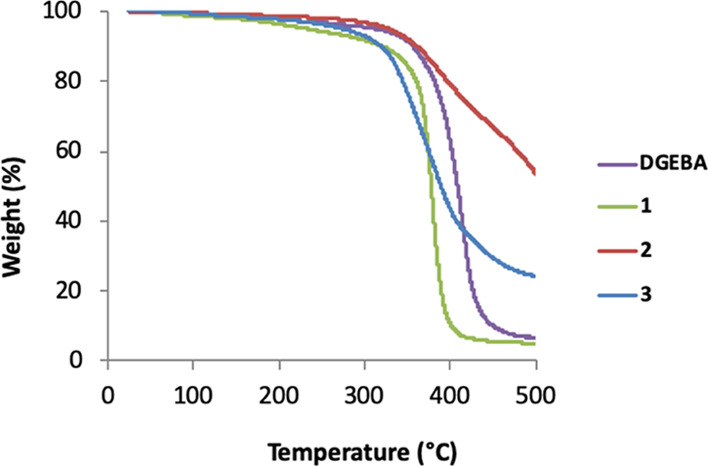
Thermogravimetric thermograms showing % weight loss at increasing temperatures of epoxy resins made from DGEBA or isosorbide bis-epoxides **1–3** in N_2_. DGEBA (purple line), **1** (green line), **2** (red line), and **3** (blue line).

Pilot experiments using TGA were conducted to investigate the properties of the polymers formed from isosorbide bis-epoxides **1–3** and the commonly used curing agent TETA (Table 2 and Figure 6). The onset of degradation is described by the initial decomposition temperature (IDT) and is an indication of the thermal stability of the cured epoxy resins. The maximum weight loss rate (*R*_max_) and the temperature (*T*_max_) at *R*_max_ were taken from the peak values of the thermograms. The activation energies (*E*_a_) for the decomposition of the polymers were calculated from the thermograms based on the Horowitz–Metzger equation.^43,44^ The polymer of **1** was found to have almost identical IDT (Table 2) and thermal degradation profile (Figure 6) as the polymer formed from DGEBA. The *T*_max_ is slightly lower and the *R*_max_ is slightly higher; however, all in all, the TGA suggests that **1** creates a polymer with very similar properties as the polymer of DGEBA. The polymer formed from **2** has very similar onset (IDT) and *T*_max_ as the DGEBA polymer (Table 2), but the *R*_max_ and the *E*_a_ are considerably lower, and the thermal degradation profile shows a much less sharp slope of the curve (Figure 6). For the polymer of **3**, the IDT was somewhat lower than that for the polymer of DGEBA, indicating lower thermal stability. In addition, all other key properties (*T*_max_, *R*_max_, and *E*_a_) for the polymer of **3** were found to be lower than those for the polymer of DGEBA (Table 2). Although not as extreme as for **2**, the polymer of **3** also displays a thermal degradation profile with a less sharp curve slope than that of the DGEBA polymer (Figure 6). This slower rate of degradation for **2** and **3** may be an indication of less-ordered polymers with a lower degree of cross-linking.

**Table 2:** Thermal Stability and Degradation Data of DGEBA and Compounds **1–3** from TGA under the Nitrogen Atmosphere

test compound	IDT^a^, °C	*T*_max_^b^, °C	*R*_max_^c^, %/°C	*E*_a_^d^, kJ/mol
DGEBA^e^	358	408	–2.17	168
**1**	362	380	–3.24	280
**2**	361	411	–0.373	66
**3**	329	382	–0.722	76

a IDT = Initial decomposition temperature which was determined with the temperature of onset of the weight loss of the sample.

b *T*_max_ = Temperature at the maximum rate of weight loss which was taken as the peak value of the differential thermogravimetric thermograms.

c *R*_max_ = Maximum weight loss rate or the slope of weight loss at *T*_max_.

d *E*_a_ = Activation energy for the decomposition of the cured epoxy resins.

e Data taken from Ponting et al.^26^

## Discussion

It is essential that the health effects of new polymeric materials are considered at the design stage prior to commercial development so that adverse effects can be minimized or prevented. The negative impact of fossil fuels on the environment has increased the interest in renewable energy sources. In this context, the massive exposure to ERS leading to ACD in workers in the wind turbine industry is a prominent example where the replacement of current ERSs with new systems with less skin-sensitizing potency is urgent to reduce the risk of skin sensitization.^15,16^

The negative impact of fossil fuels has also sparked an increased interest in renewable biobased starting materials for polymers. We have previously explored the possibility to use the plant lignan pinoresinol as a starting material for the production of less allergenic and nature-derived epoxy resin monomers.^45^ The synthesized ERM, pinoresinol diglycidyl ether (PinoDGE), was found to be nonsensitizing at all the tested concentrations (up to 0.17 M) in the LLNA, which is in marked difference to DGEBA and DGEBF that classify as strong sensitizers. Our preliminary investigation of the polymer formed from PinoDGE was also promising, but unfortunately, pinoresinol was found to be too costly to proceed to large-scale production. Isosorbide is a byproduct from the starch industry, produced by catalytic hydrogenation of d-glucose, which yields d-sorbitol, followed by double dehydration. This sugar-based cycloaliphatic diol is a green and renewable resource that, in contrast to pinoresinol, holds potential as a price-competitive starting material for large-scale production of the next generation of thermosetting resins.^46^ Isosorbide is classified as safe by the European Food Safety Authority (EFSA)^47^ and it has been shown to have no endocrine effects.^48^ We therefore identified isosorbide as a promising starting material for the synthesis of bis-epoxide ERMs with less sensitizing potencies. This would be a clear advantage over petroleum-derived DGEBA, currently used in large quantities as an ERM, which is harmful both as a skin sensitizer^4−8^ and as an endocrine disruptor.^49^

In addition to the diglycidyl ether of isosorbide (**1**), two larger isosorbide-based bis-epoxides containing either a benzoic acid ester (**2**) or a benzyl ether (**3**) linkage were synthesized (Schemes 1^2^,3). A compound similar to **2** is described in the literature,^46,50^ isosorbide bis(4-glycidyl benzoate), with the only difference being that we have removed the glycidyl ether oxygen as we have shown in previous investigations that this modification lowers the sensitizing potency of ERMs.^23,24^ The in vivo assessments of the sensitizing potency of isosorbide bis-epoxides **1–3** (Figure 2 and Table S1) were in agreement with our hypothesis as **2** and **3** were found to be less sensitizing than **1** (which has a glycidyl ether oxygen). The new compounds all showed lower sensitizing potencies compared to DGEBA which is a strong sensitizer (EC3 = 0.036 M).^24^ We identified **1** and **3** as moderate sensitizers (EC3 = 0.17 M and 0.36 M, respectively) while **2** was nonsensitizing in concentrations up to 25% w/v (0.54 M) (Figure 2 and Table S1). This can be compared to our previous studies on the sensitizing potency of structural analogues of DGEBA/F, in which the DGEBF analogue that lacks the glycidyl ether oxygen displayed a three times decrease in sensitizing potency (EC3 = 0.091 M, 2.56% w/v) in vivo compared to DGEBA/F.^24^ In a following study, we found that the sensitizing potency was decreased 10 times compared to DGEBA/F (EC3 = 0.38 M, 12% w/v) by removing both the aromaticity and the glycidyl ether oxygen.^26^

Most haptens, including DGEBA and analogues thereof, are electrophilic compounds that can modify proteins by forming covalent bonds with their nucleophilic moieties such as cysteines (thiols) and lysines (primary amines). Therefore, peptide reactivity is often used as a nonanimal-based tool to assess the skin-sensitizing potency of a compound.^51,52^ In our previous work, we have seen good correspondence between the sensitizing potency of DGEBA analogues in the LLNA and their reactivity toward the cysteine moiety of the peptide AcPHCKRM.^22−24,42^ Also, in this study, the order of peptide depletion for the three investigated isosorbide bis-epoxides **1–3** agreed with their sensitizing potency in the LLNA as **1** is the most reactive in both assays, followed by **3**, and the least reactive is **2** (Figures 2 and 3). The peptide reactivity experiments as well as computational studies support the finding that **2** is less reactive than **1**, probably due to the lack of glycidyl ether oxygen. The finding that **2** is less reactive than **3** (in both of these assays) is believed to be because of the less electron-rich aromatic ring system of **2**.^26^

To investigate if a difference in skin permeation is a contributing factor to the lower in vivo sensitizing potency of isosorbide bis-epoxides **2** and **3**, they were compared to DGEBA in a permeability experiment based on the OECD Test Guidelines^35^ that utilizes a static Franz diffusion cell and pig skin. The detected amounts of all compounds in the receptor fluid were in a similar range (Table 1, Figure 4). This was also the case after the permeability experiment when the skin was extracted overnight to determine free amounts on/in the skin. Thus, the permeability experiment did not provide conclusive explanations to the observed differences in sensitizing potency.

Since we considered bis-epoxides **1–3** to be promising starting points for the development of biobased, less sensitizing alternatives to DGEBA, we conducted preliminary investigations of the properties (Table 2) of the polymers obtained after polymerization of **1–3** with TETA. **1** showed very similar polymer properties as DGEBA both regarding the IDT and polymer decomposition profile (Table 2 and Figure 6). Polymers based on **1** have previously been evaluated by others, and although these polymers were found to have excellent dry mechanical properties, similar to polymers of DGEBA, the glass transition temperature (*T*_g_) of the polymers based on **1** was found to be significantly lower than that of DGEBA polymers.^29,53^ This decrease in *T*_g_ was assigned to the high hydrophilicity of polymers of **1**.^29,53^ Jaffe and co-workers concluded that polymers of **1** might be useful as ERM for biodegradable products such as drug delivery systems but not for thermosetting resins, which require higher *T*_g_ and lower hydrophilicity.

We found that bis-epoxide **3** had a lower IDT and slower polymer decomposition rate than DGEBA, whereas bis-epoxide **2** had a similar IDT as DGEBA but a slower polymer decomposition rate (Table 2 and Figure 6). **2** is structurally very similar to isosorbide glycidyl benzoate synthesized by Jaffe and co-workers,^46^ with the only exception being that **2** lacks glycidyl ether oxygen. Jaffe and co-workers found that isosorbide bis(4-glycidyl benzoate) had a *T*_g_ comparable to, or even higher than, the *T*_g_ of DGEBA polymers and that the hydrophobicity was significantly increased compared to polymers of **1**. Hence, they concluded that isosorbide glycidyl benzoate has the potential to replace DGEBA in the production of thermosetting resins for food packaging and surface coating.^46^ Thus, one could hypothesize that the wet properties of polymers from **2** would also be technically satisfactory. As **2** was found to be nonsensitizing in concentrations up to 25% w/v, it does have great potential as a renewable and less sensitizing replacement of DGEBA.

In conclusion, all three isosorbide bis-epoxides **1–3** have the potential to be interesting as replacements of DGEBA and should be investigated further concerning their technical properties. **2** appears to be the most promising of the investigated isosorbide bis-epoxides, as it displayed the lowest sensitizing potency in vivo as well as the lowest peptide reactivity. Taking health effects into account, our study has shown that glucose-based isosorbides are interesting alternatives to the presently used petroleum-based ERMs as raw materials for the preparation of polymers in various applications.

## Supporting Information Available

Detailed LLNA information; MS fragment assignment from the peptide depletion experiments; raw data from skin permeability experiments of DGEBA, **2** and **3**; procedures for polymerization and thermogravimetric analysis; and NMR spectra for compounds **1a**, **1**, **2a**, **2b**, **2**, **3a**, and **3** (PDF)

## Abbreviations

ACD: allergic contact dermatitis
BPA: bisphenol A
CDI: 1,1′-carbonyldiimidazole
*m*-CPBA: 3-chloroperbenzoic acid
DFT: density functional theory
DGEBA: diglycidyl ether bisphenol A
DMSO: dimethyl sulfoxide
ESI: electrospray ionization
ERM: epoxy resin monomer
ERS: epoxy resin system
LLNA: local lymph node assay
LOQ: limit of quantification
MW: microwave heating
PBS: phosphate buffered saline
SI: stimulation index
TBAB: tetrabutylammonium bromide
THF: tetrahydrofuran
TGA: thermogravimetric analysis
TLC: thin-layer chromatography
TETA: triethylenetetramine
