# Supplementary material for: Nature-Derived Epoxy Resin Monomers with Reduced Sensitizing Capacity—Isosorbide-Based Bis-Epoxides
Source: Chem Res Toxicol. 2023 Jan 18;36(2):281–90. doi: 10.1021/acs.chemrestox.2c00347 (PMC9945177; doi:10.1021/acs.chemrestox.2c00347)
Supplement: Supplementary file 1 — tx2c00347_si_001.pdf [file tx2c00347_si_001.pdf]

Supporting Information for

Nature Derived Epoxy Resin Monomers with Reduced Sensitizing Capacity – Isosorbide-based Bis-epoxides

Isabella Karlsson,[†,] David J. Ponting,[‡] Miguel A. Ortega,[‡] Ida B. Niklasson,[‡] Lorena Ndreu,[†] E. Johanna L. Stéen,[‡] Tina Seifert,[§] Kristina Luthman[§] and Ann-Therese Karlberg[‡]*

[†]Department of Environmental Science, Exposure and Effect, Stockholm University, SE-106 91 Stockholm, Sweden

[‡]Department of Chemistry and Molecular Biology, Dermatochemistry and Skin Allergy, University of Gothenburg, SE-412 96 Gothenburg, Sweden

[§]Department of Chemistry and Molecular Biology, Medicinal Chemistry, University of Gothenburg, SE-412 96 Gothenburg, Sweden

** Corresponding author. E-mail: Isabella.Karlsson@aces.su.se*

Table of contents

Local Lymph Node Assay 3

Experimental Protocol 3

Results from the Local Lymph Node Assay 4

Table S1: Full results from the LLNA of 1-3 4

Fragment Assignments for Peptide Reactivity Experiments 5

Figure S1. Fragment assignments for Ac-Pro-His-Cys-Lys-Arg-Met (AcPHCKRM) 5

Skin Permeability Experiments 6

Table S2: UPLC-ESI-MS/MS parameters used for quantification of the different compounds 6
Table S3: Raw data for DGEBA 6
Table S4: Raw data for isosorbide bis-epoxide **2** 6
Table S5: Raw data for isosorbide bis-epoxide **3** 6

Polymerization Procedure and Thermogravimetric Analysis 6

Polymerization Procedure 7

Thermogravimetric Analysis (TGA) 7

NMR Spectra of Synthesized Compounds 8

^{1}H NMR for compound 1a 8
^{1}H and ^{13}C NMR for compound 1 9
^{1}H and ^{13}C NMR for compound 2a 10
^{1}H and ^{13}C NMR for compound 2b 11
^{1}H and ^{13}C NMR for compound 2 12
^{1}H and ^{13}C NMR for compound 3a 13
^{1}H and ^{13}C NMR for compound 3b 14
^{1}H and ^{13}C NMR for compound 3 15

References 16

Local Lymph Node Assay

Experimental Protocol

A previously published protocol[1] of the LLNA[2] was used to assess the sensitization potential of the isosorbide bis-epoxides **1-3**. The purity of the assessed isosorbide bis-epoxides was >98% according to LC/MS analysis. The isosorbide bis-epoxides were tested in five different concentrations; **1**: 3.87 mM (0.10% w/v), 38.7 mM (1.0% w/v), 194 mM (5.0% w/v), 387 mM (10% w/v), and 774 mM (20% w/v); **2**: 21.4 mM (1.0% w/v), 53.6 mM (2.5 % w/v), 107 mM (5.0% w/v), 214 mM (10% w/v), and 536 mM (25% w/v); and **3**: 2.28 mM (0.1% w/v), 22.8 mM (1.0% w/v), 114 mM (5.0% w/v), 228 mM (10% w/v), and 456 mM (20% w/v). Briefly, mice in groups of three received 25 µL of test compound dissolved in the vehicle acetone/olive oil (4:1 v/v), on the dorsum of the ears for three consecutive days (day 0, 1, and 2). A sixth group of four mice was treated with vehicle only (control group). On day 5, all mice were injected intravenously via the tail vein with 250 µL of phosphate buffered saline (PBS) (pH 7.4, 137 mM NaCl, 2.7 mM KCl, and 10 mM phosphate buffer) containing 20 µCi [methyl-^{3}H]thymidine. Five hours later the mice were sacrificed, the draining lymph nodes were excised and pooled for each group, followed by preparation of single-cell suspensions of lymph nodes in PBS by using cell strainers (Falcon; BD Labware, Franklin Lakes, NJ, USA; pore size, 70 µm). Cell suspensions were washed twice with PBS, precipitated with trichloroacetic acid (TCA) (5%), and stored at 4 °C overnight. The samples were then centrifuged, resuspended in TCA (5%, 1 mL), and transferred to scintillation cocktail (10 mL) (EcoLume INC Radiochemicals, Solon, OH, USA). The incorporation of [methyl-^{3}H]thymidine into DNA was measured by β-scintillation counting with a Beckman LS 6000TA instrument. Results are expressed as the mean dpm/lymph node for each experimental group and as stimulation index (SI). The SI is defined as the ratio between dpm/lymph node for the test group and the control group. Test materials that at one or more concentrations cause an SI greater than 3 are considered positive in the LLNA. EC3 values (the estimated concentration required to induce an SI of 3) were calculated by linear interpolation. The sensitizing potency was classified as follows: EC3 $\leq$ 0.2%, extreme; 0.2% < EC3 $\leq$ 2%, strong; EC3 > 2%, moderate.[3]

Results from the Local Lymph Node Assay

Table S1: Full results from the LLNA of bis-epoxides **1-3**[a]

Compound	Test concentration (% w/v)	Test concentration (M)	[^{3}H]thymidine incorporation (dpm/lymph node)	SI	EC$_3$ value (% w/v)	EC$_3$ value (M)
**1**	Control		260		4.40	0.17
	0.10	3.9 × 10^{-3}	213	0.82		
	1.0	39 × 10^{-3}	367	1.41		
	5.0	0.19	852	3.28		
	10	0.39	3164	12.2		
	20	0.78	4121	15.8		
**2**	Control		429		n/a	n/a
	1.0	21 × 10-3	455	1.06		
	2.5	54 × 10^{-3}	614	1.43		
	5.0	0.11	473	1.10		
	10	0.21	459	1.07		
	25	0.54	972	2.27		
**3**	Control		453		15.8	0.36
	0.10	2.3 × 10^{-3}	479	1.06		
	1.0	23 × 10^{-3}	706	1.56		
	5.0	0.11	1158	2.55		
	10	0.23	1292	2.85		
	20	0.46	1412	3.11		

[a] Groups of mice were treated with the test substance in five different concentrations, on the dorsum of both ears for three consecutive days. Sham treated control animals received the vehicle alone. On day five, all mice were injected intravenously with PBS (250 μL) containing 20 μCi of [methyl-^{3}H]thymidine. After 5 h the mice were sacrificed, the draining lymph nodes were excised and pooled for each group, single cell suspensions of lymph-node cells were prepared, and the thymidine incorporation into DNA was measured by β-scintillation counting. The increase in thymidine incorporation relative to vehicle-treated controls was derived for each experimental group and recorded as stimulation index (SI). The EC$_3$ value (the estimated concentration required to induce an SI of 3) was calculated using linear interpolation.

Fragment Assignments for Peptide Reactivity Experiments

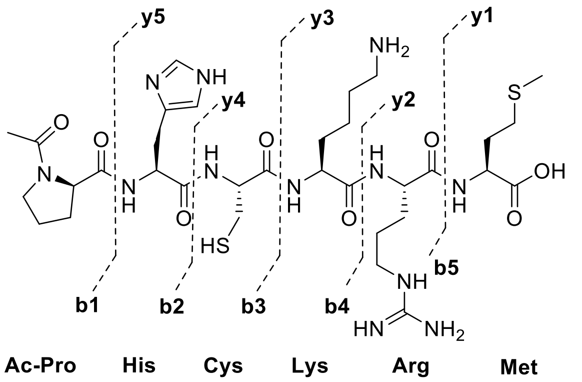

Molecular ion mass:
MH^+: 813.2
b- and y- fragment masses:

b1: 140.1	y1: 150.1
b2: 277.1	y2: 306.2
b3: 380.1	y3: 434.2
b4: 508.2	y4: 537.3
b5: 664.3	y5: 674.3

Figure S1. Fragment assignments for Ac-Pro-His-Cys-Lys-Arg-Met (AcPHCKRM)
The haptenated positions on the peptide were determined by analysis of the fragmentation pattern of the mass spectra of conjugates formed between compound and peptide. Peptides are known to form specific fragments by collision-induced decomposition (CID).[4] The most common fragments observed in the mass spectra of AcPHCKRM conjugates were b- and y-fragments.[5]

Skin Permeability Experiments

Table S2: UPLC-ESI-MS/MS parameters used for quantification of the different compounds

Test compound	Parent Ion (m/z)	Retention Time (min)	Cone Voltage (V)	Transition (m/z)	Collision Energy (V)
DGEBA	341.16	8.84	30	341.16 > 133	15
				341.16 > 173	15
**2**	467.16	8.15	30	467.16 > 105	30
				467.16 > 161	17
**3**	439.26	7.62	35	439.26 > 69	20
				439.26 > 131	12

Table S3: Raw data for DGEBA

Timepoint (h)	Replicate 1 (µg/ml)	Replicate 2 (µg/ml)	Replicate 3 (µg/ml)	Average (µg/ml)	Average (µM)	STD[a] (µg/ml)	STD[a] (µM)
0	<LOQ[b]	<LOQ[b]	<LOQ[b]	0	0	0	0
1	<LOQ[b]	<LOQ[b]	<LOQ[b]	0	0	0	0
2	0.315	0.184	0.108	0.203	0.595	0.0856	0.251
3	0.419	0.239	0.177	0.278	0.818	0.103	0.303
4	0.456	0.229	0.202	0.296	0.868	0.114	0.334
5	0.436	0.237	0.196	0.290	0.851	0.105	0.307
6	0.406	0.218	0.210	0.278	0.817	0.0904	0.265
Extract	0.0658	<LOQ[b]	0.0742	0.0700	0.206	0.00416	0.0122

[a] Standard deviation (STD); Limit of quantification (LOQ) was determined by analysis of the analyte at low concentration and was calculated as 10 times the signal-to-noise ratio.

Table S4: Raw data for isosorbide bis-epoxide **2**

Timepoint (h)	Replicate 1 (µg/ml)	Replicate 2 (µg/ml)	Replicate 3 (µg/ml)	Average (µg/ml)	Average (µM)	STD[a] (µg/ml)	STD[a] (µM)
0	<LOQ[b]	<LOQ[b]	<LOQ[b]	0	0	0	0
1	0.0637	0.0511	0.574	0.0574	0.123	0.00515	0.0110
2	0.299	0.233	0.154	0.228	0.490	0.0592	0.127
3	0.437	0.320	0.180	0.312	0.669	0.105	0.226
4	0.538	0.362	0.213	0.371	0.796	0.133	0.284
5	0.500	0.323	0.186	0.336	0.721	0.128	0.275
6	0.474	0.309	0.166	0.317	0.679	0.126	0.270
Extract	0.203	0.139	0.141	0.161	0.345	0.0297	0.0636

[a] Standard deviation (STD); Limit of quantification (LOQ) was determined by analysis of the analyte at low concentration and was calculated as 10 times the signal-to-noise ratio.

Table S5: Raw data for isosorbide bis-epoxide **3**

Timepoint (h)	Replicate 1 (µg/ml)	Replicate 2 (µg/ml)	Replicate 3 (µg/ml)	Average (µg/ml)	Average (µM)	STD[a] (µg/ml)	STD[a] (µM)
0	<LOQ[b]	<LOQ[b]	<LOQ[b]	0	0	0	0
1	0.0897	0.110	0.0460	0.0818	0.187	0.0266	0.0606
2	0.139	0.292	0.180	0.204	0.465	0.0648	0.148
3	0.191	0.347	0.203	0.247	0.563	0.0709	0.161
4	0.202	0.309	0.203	0.238	0.542	0.0504	0.115
5	0.204	0.306	0.198	0.236	0.539	0.0493	0.112
6	0.165	0.298	0.189	0.217	0.495	0.0578	0.132
Extract	0.0650	0.000965	0.0309	0.0323	0.0736	0.0262	0.0597

[a] Standard deviation (STD); Limit of quantification (LOQ) was determined by analysis of the analyte at low concentration and was calculated as 10 times the signal-to-noise ratio.

Polymerization Procedure and Thermogravimetric Analysis

Polymerization Procedure

The polymerization reaction between the ERMs DGEBA and compounds **1-3** with TETA was performed as previously described.[1] The bis-epoxide and TETA were thoroughly mixed for 2 min at room temperature, always at a one-to-two ratio of epoxy group to amino-hydrogens, and thereafter transferred to cavities of silicon embedding mold and placed in a vacuum desiccator. The mixture was degassed under vacuum for 10 min to remove acetone and trapped air. The cast resin was then cured at room temperature for 24 h followed by postcuring in an oven at 120 °C for 2 h. After the postcure, the oven was switched off and allowed to cool slowly to room temperature to avoid crack formation.

Thermogravimetric Analysis (TGA)

The TGA analyses were performed on polymers prepared from the bis-epoxides **1**-**3** using a Perkin–Elmer TGA 7 or a NETZSCH TG 209 instrument under nitrogen purge. The polymers were heated from 25 to 500 °C at a rate of 10 °C/ min and the weight loss was determined for each sample as a function of temperature.

NMR Spectra of Synthesized Compounds

^{1}H NMR for **1a**

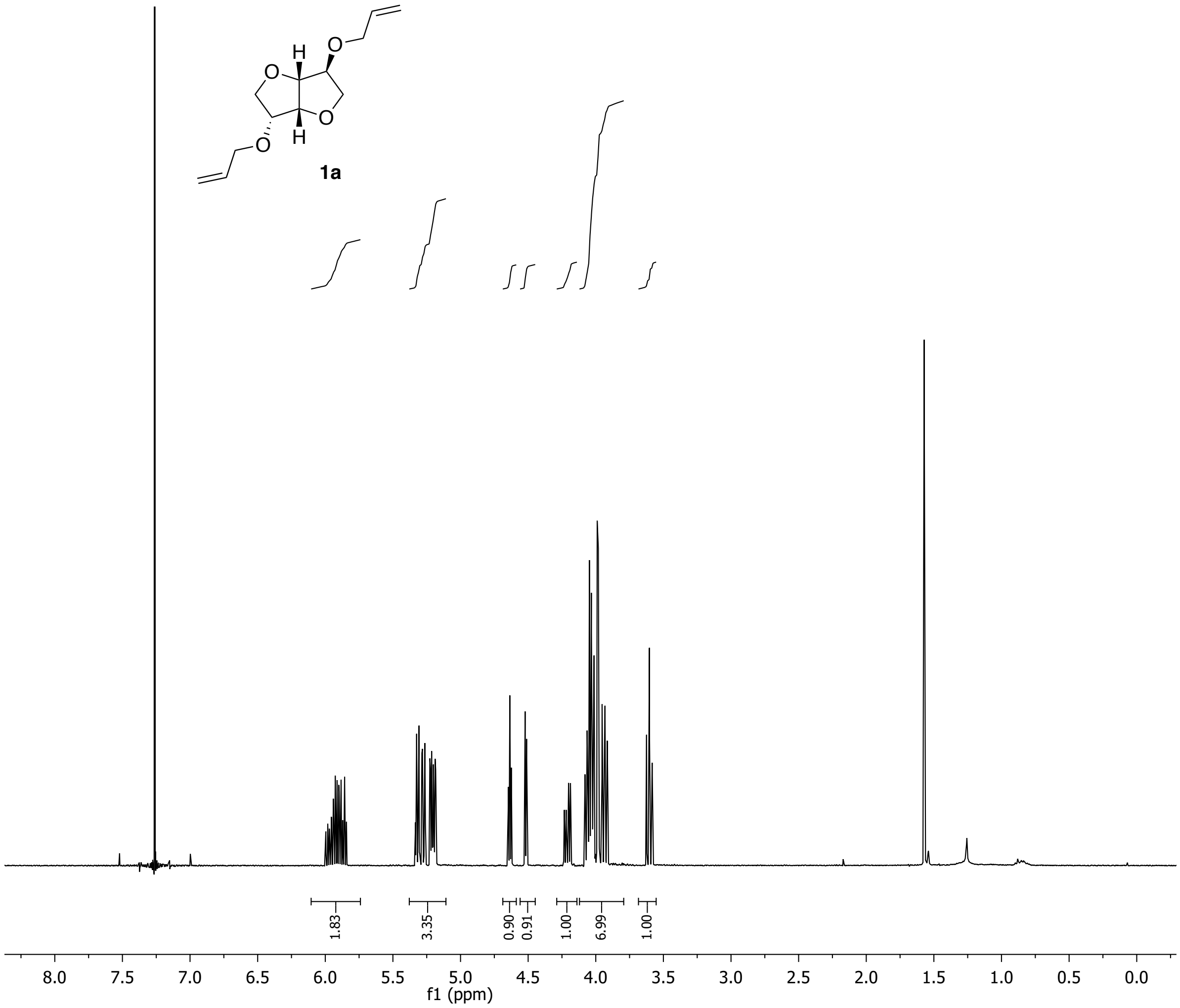

^{1}H and ^{13}C NMR for **1**

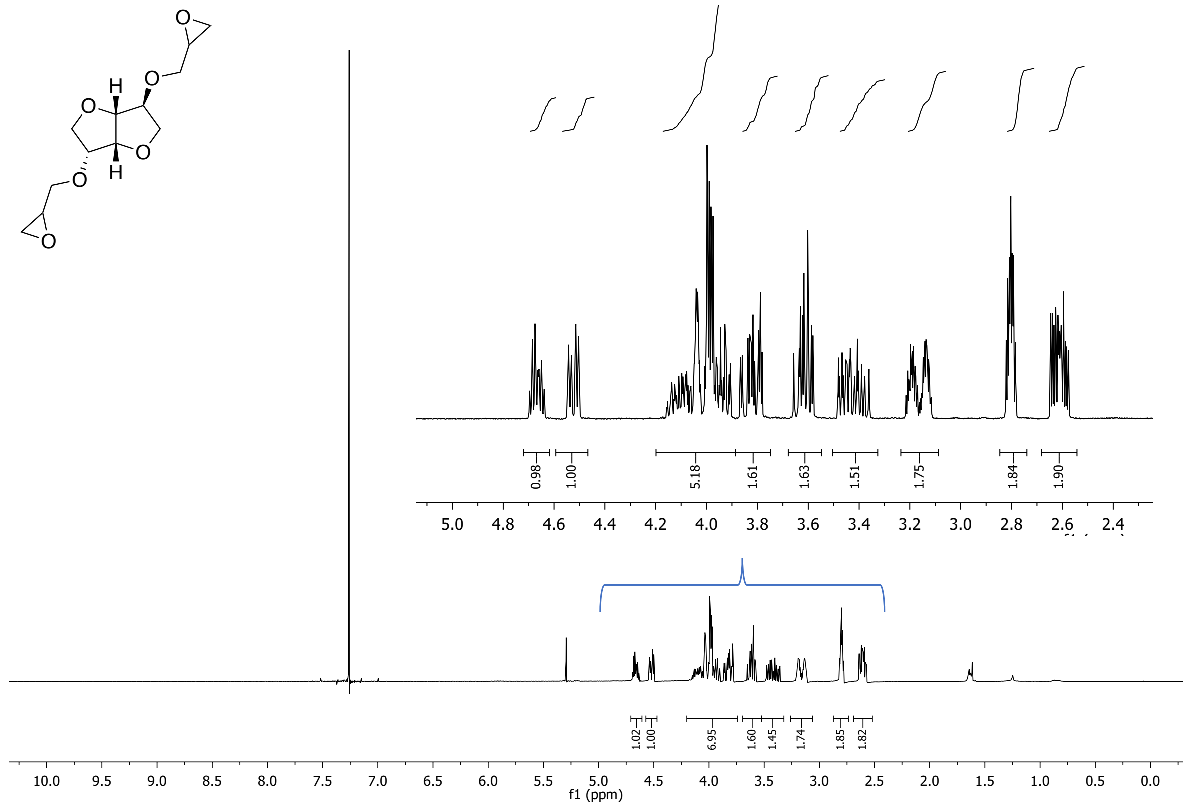

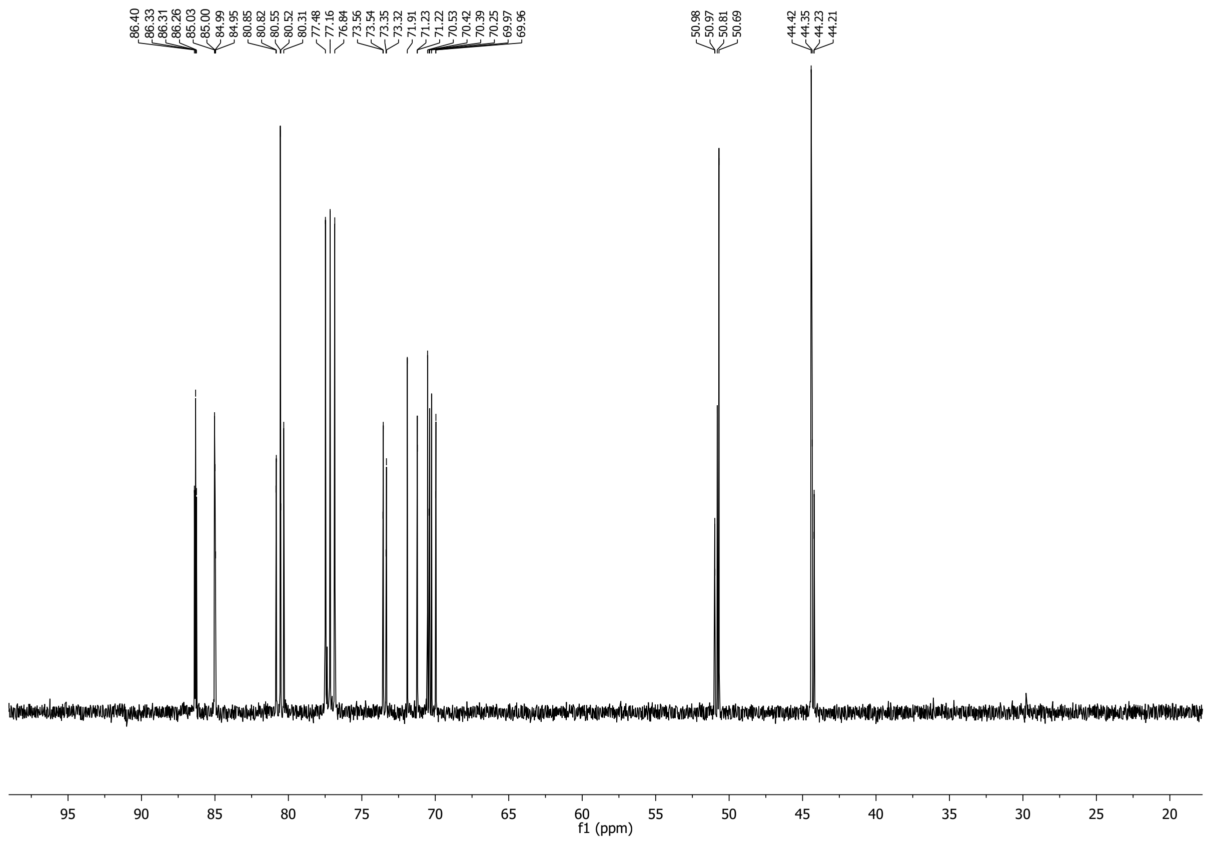

^{1}H and ^{13}C NMR for **2a**

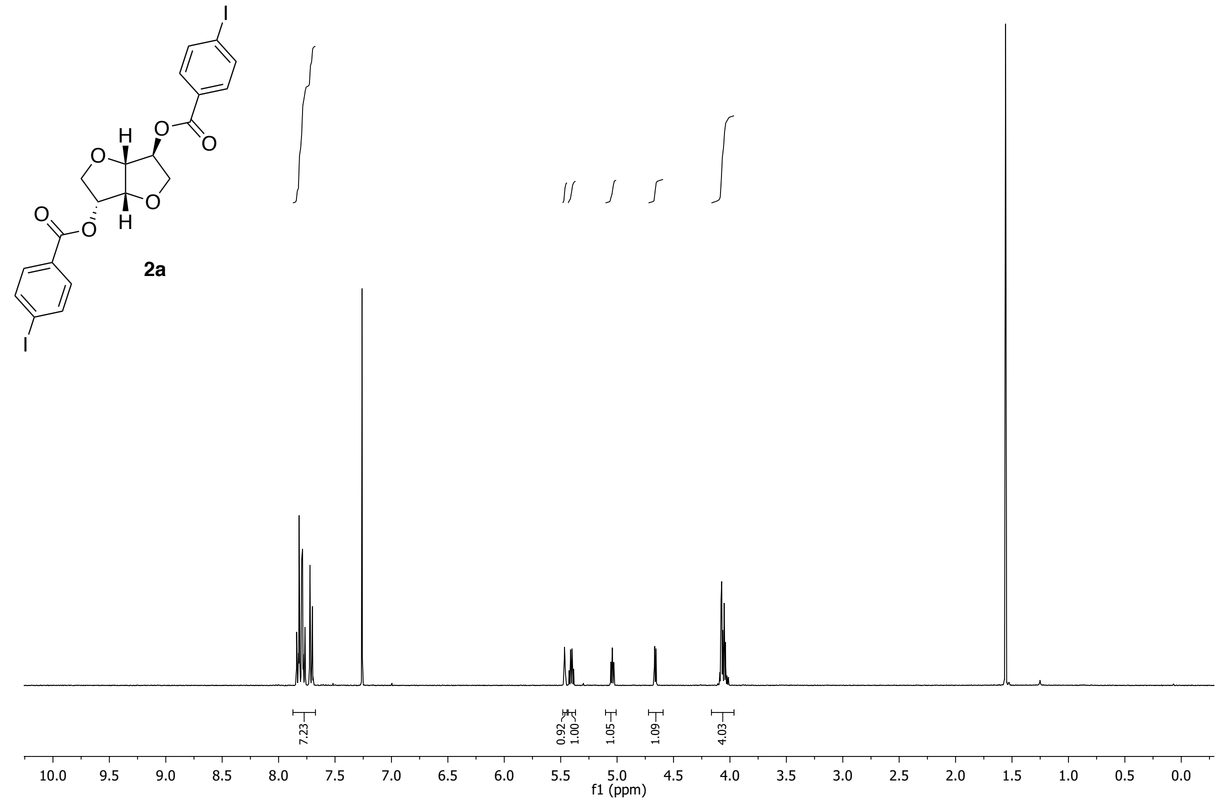

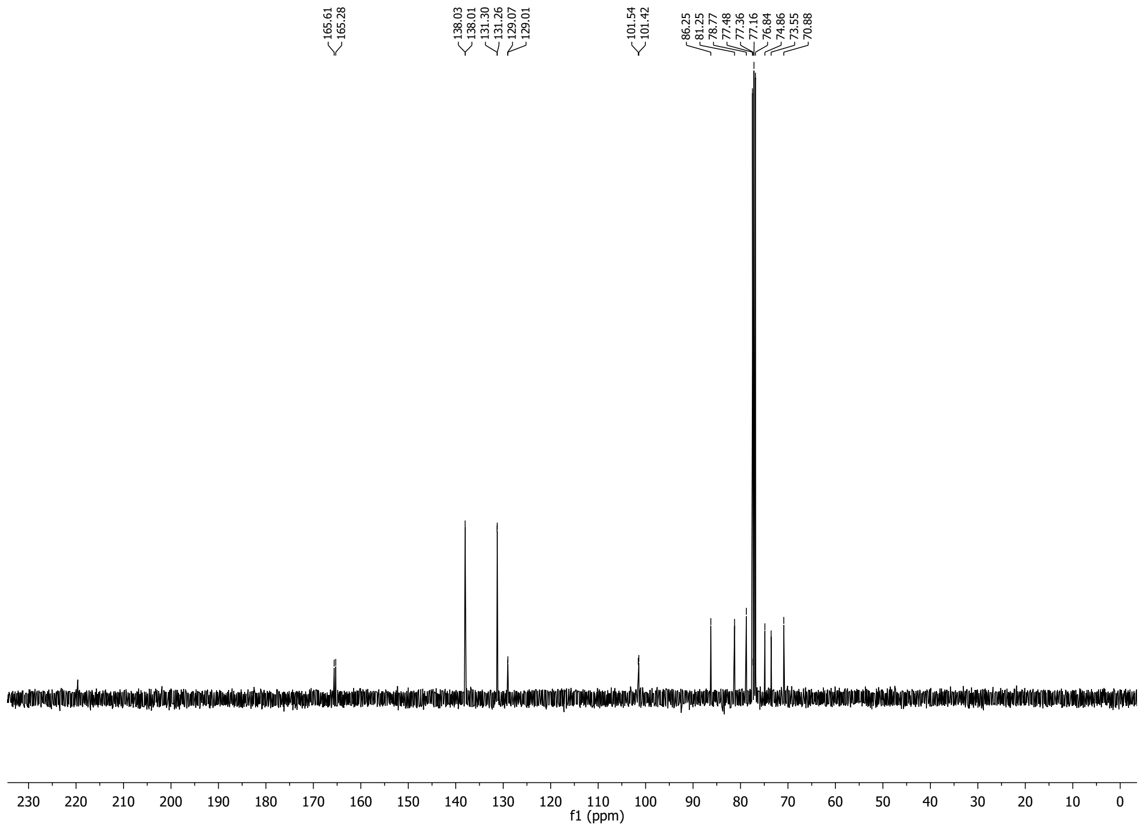

^{1}H and ^{13}C NMR for **2b**

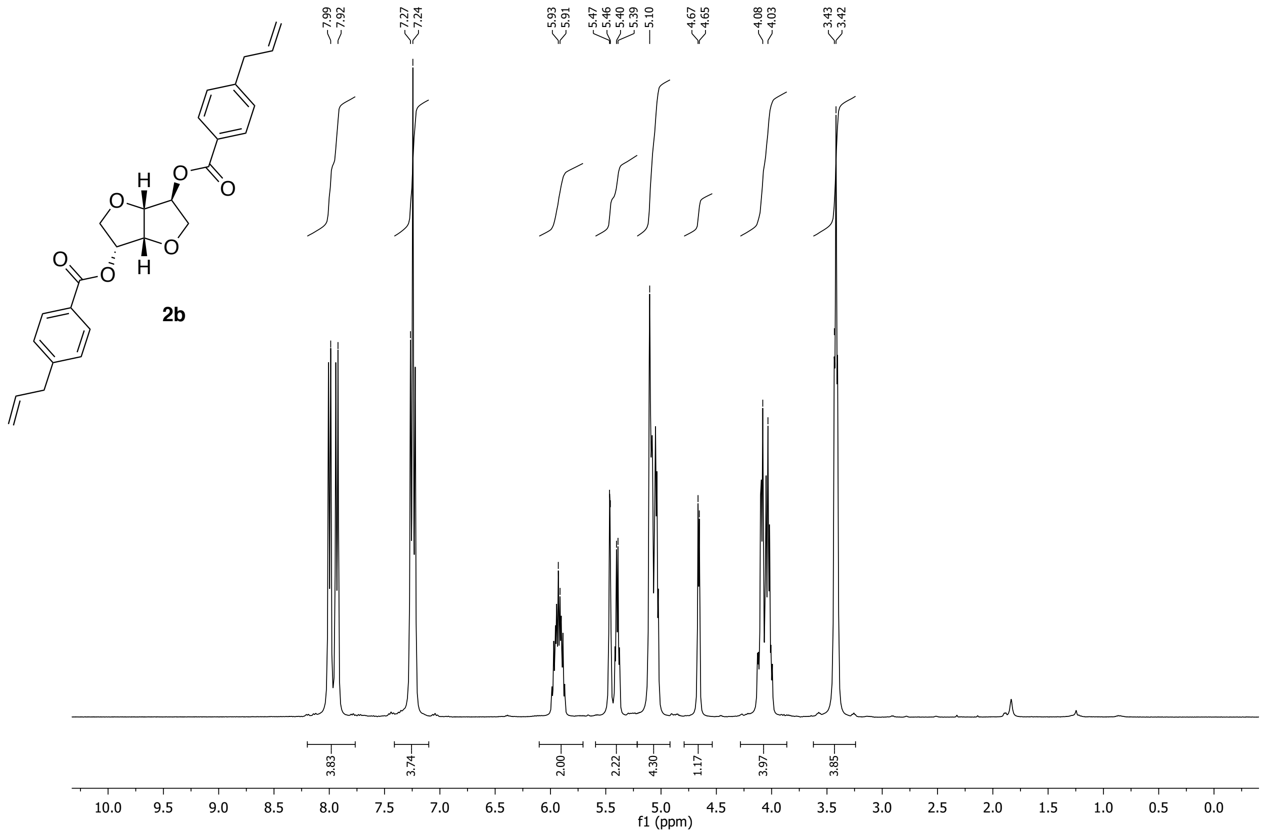

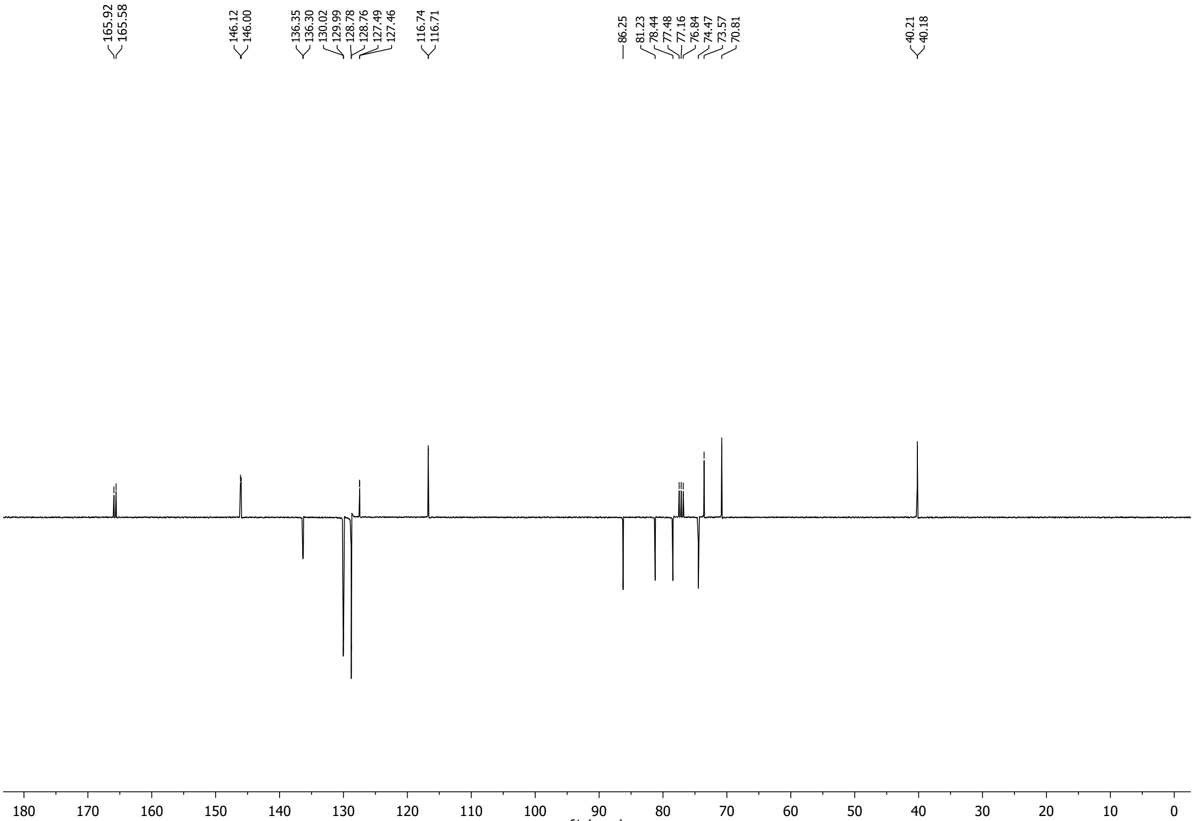

^{1}H and ^{13}C NMR for **2**

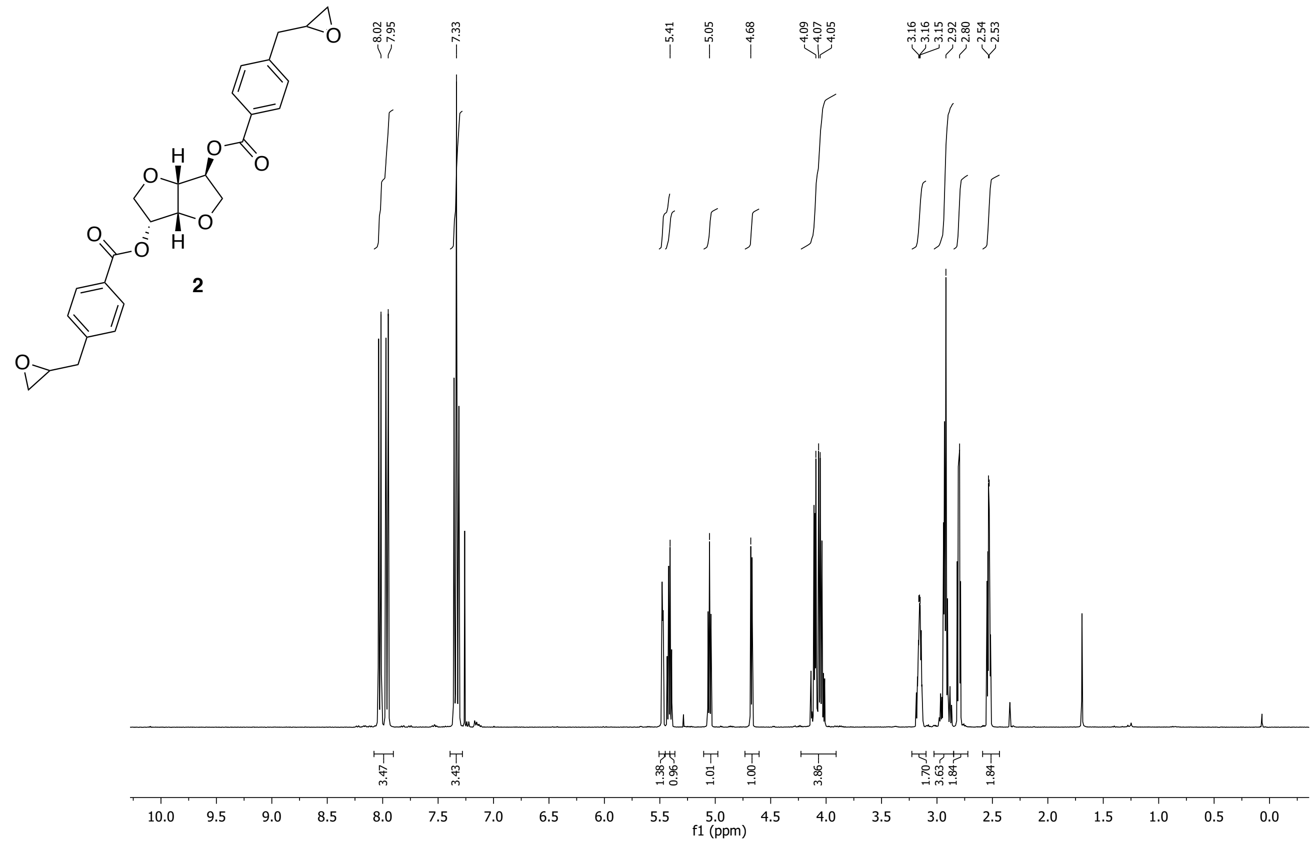

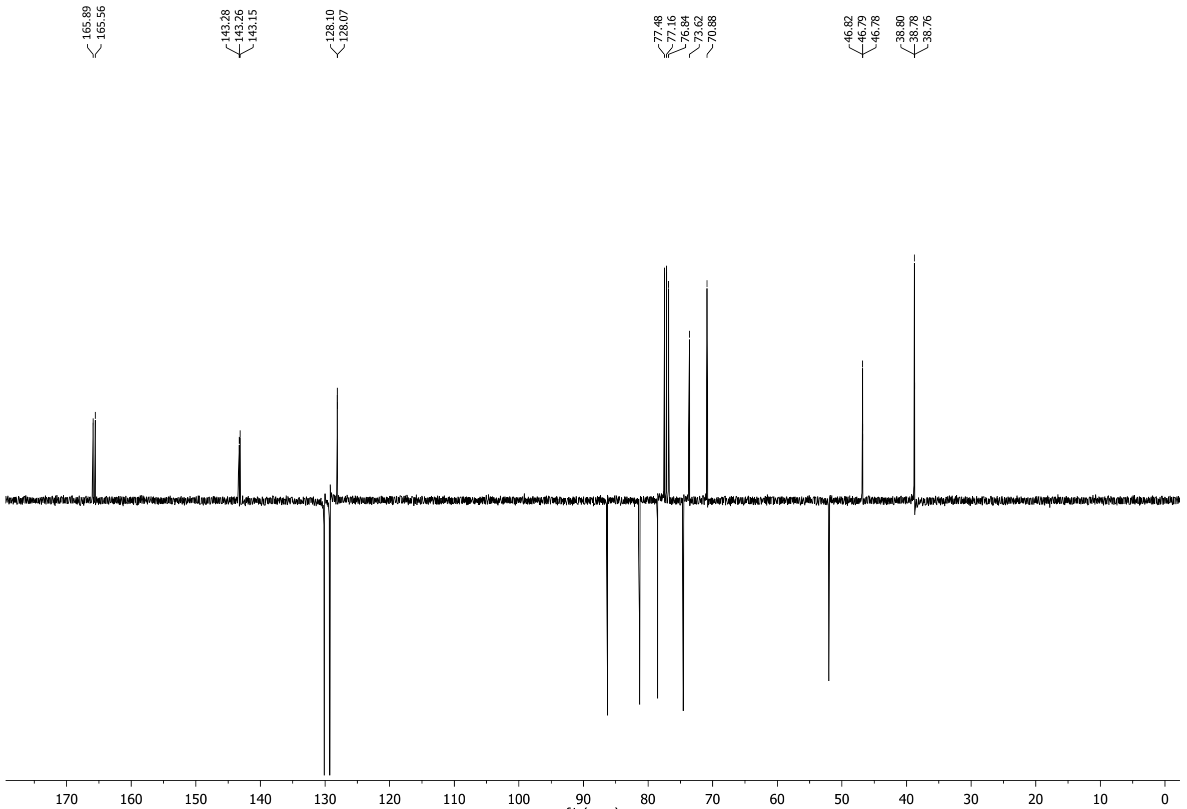

^{1}H and ^{13}C NMR for **3a**

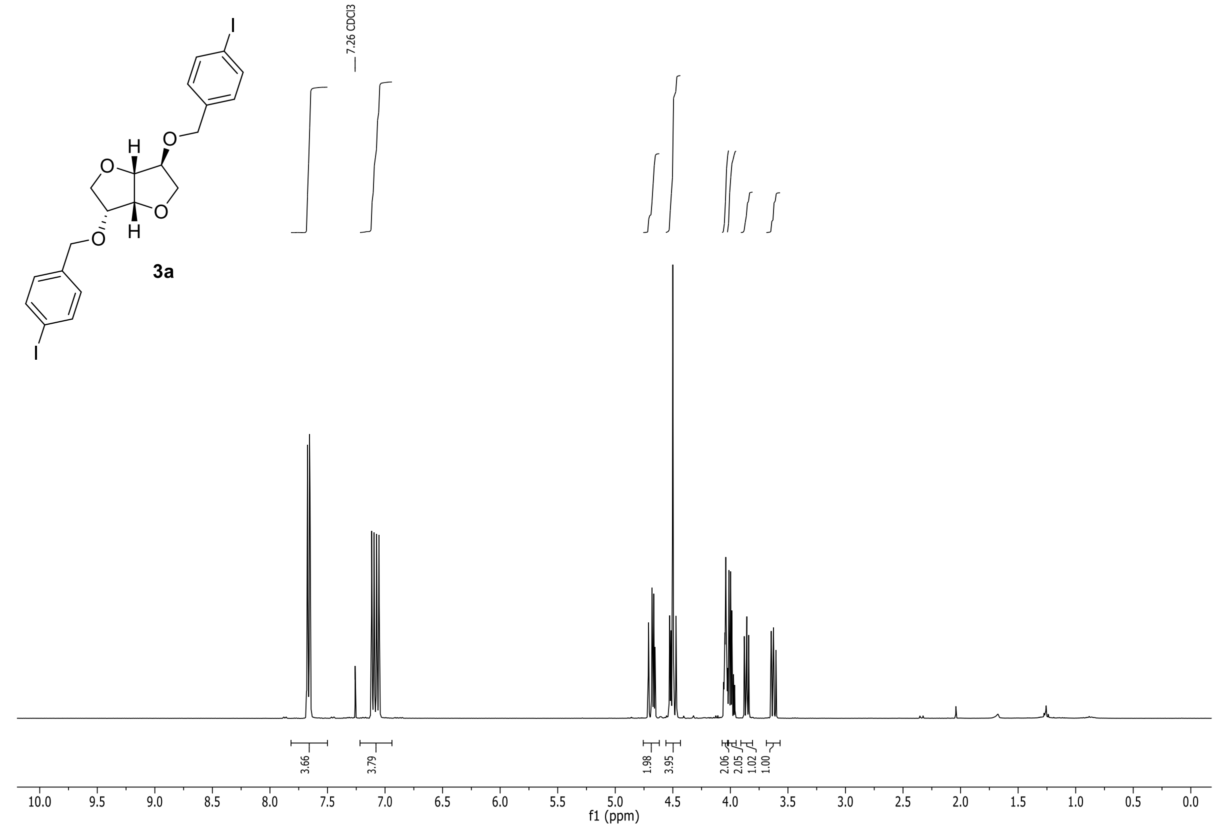

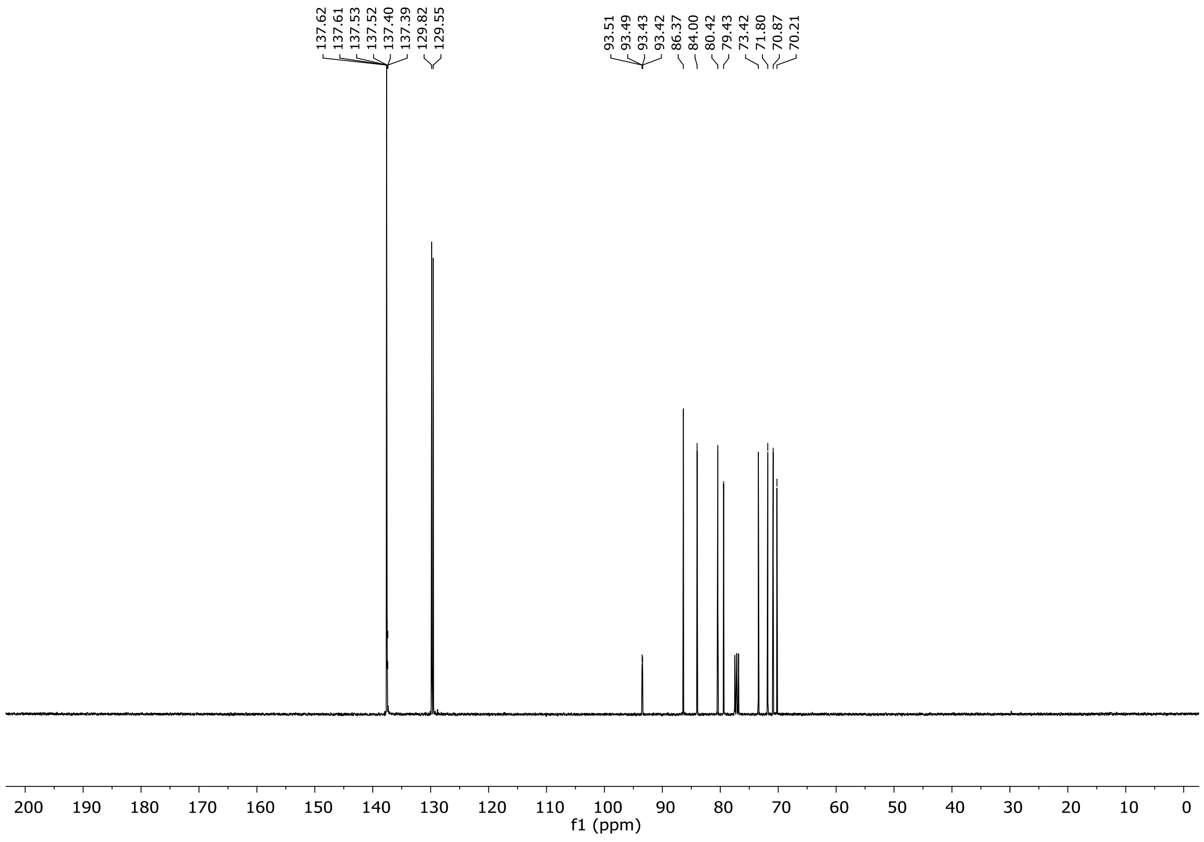

^{1}H and ^{13}C NMR for **3b**

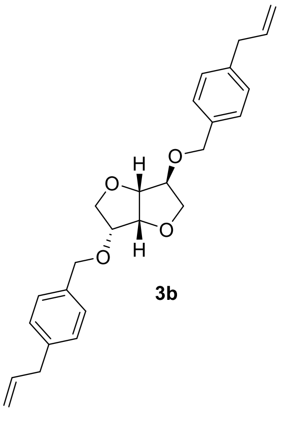

3b

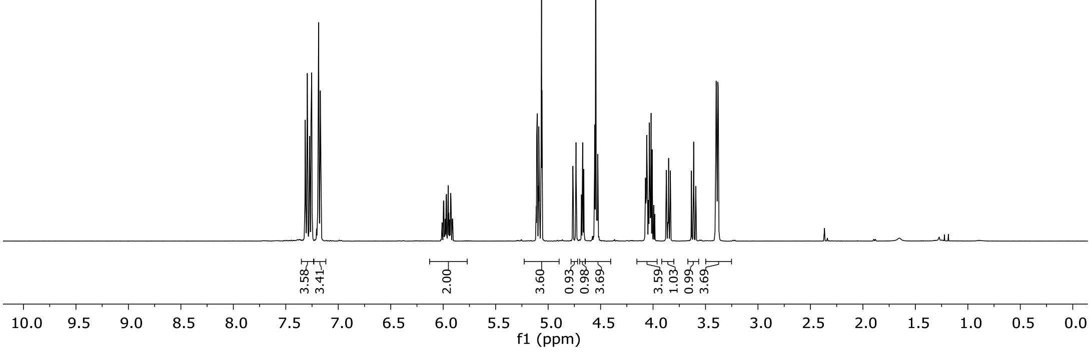

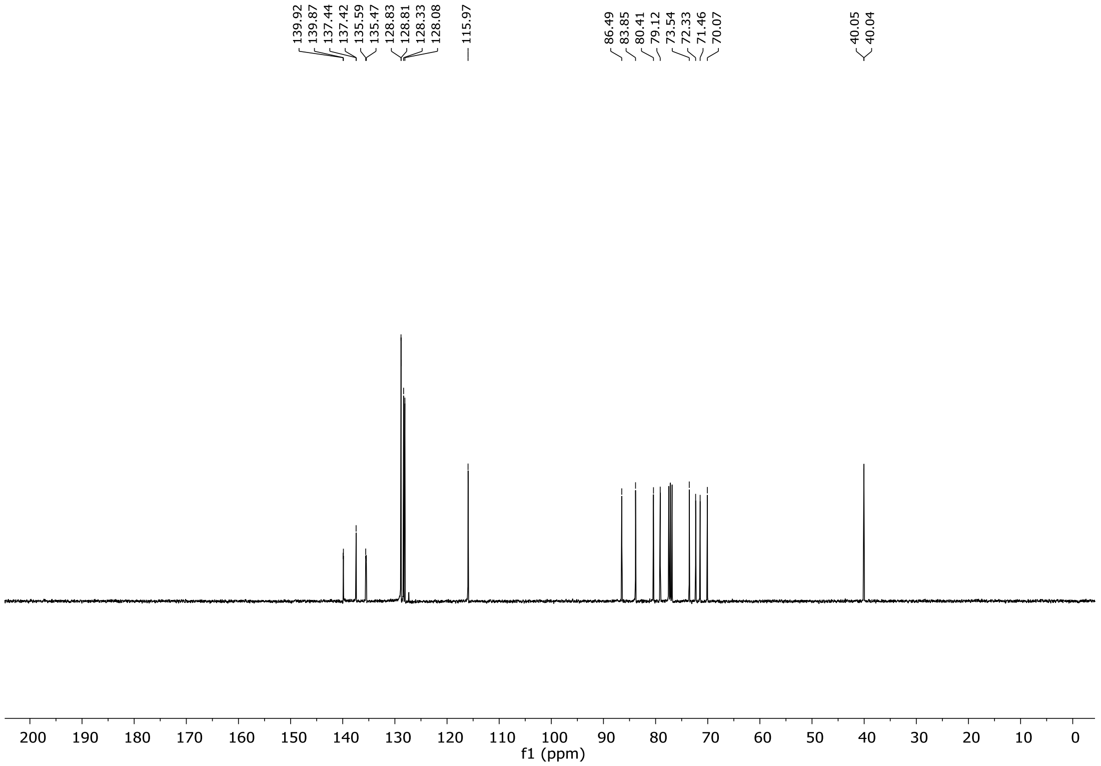

^{1}H and ^{13}C NMR for **3**

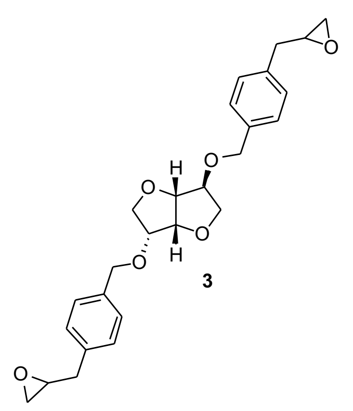

3

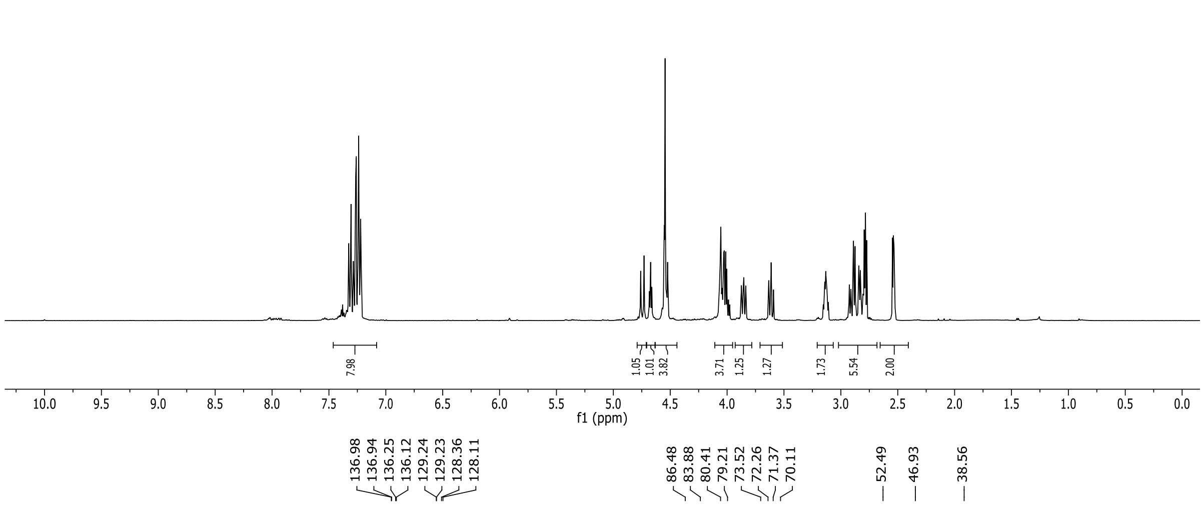

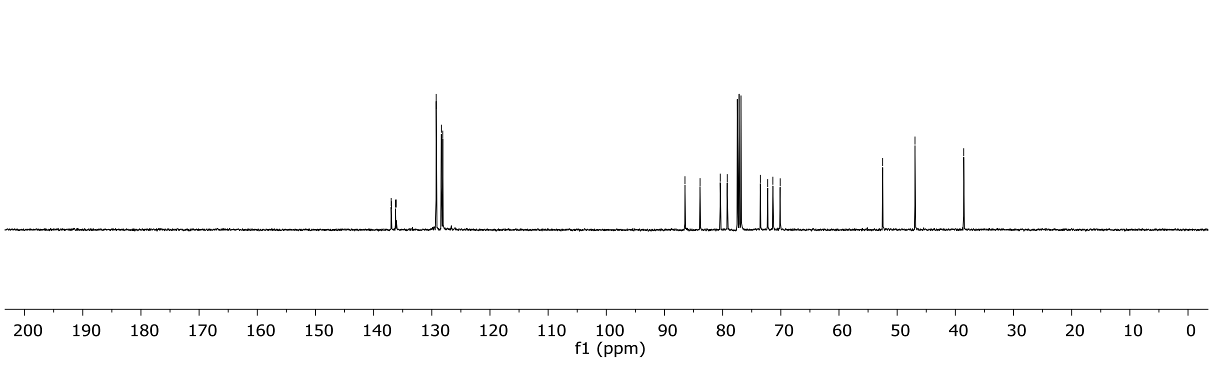

References

(1) O'Boyle, N. M., Niklasson, I. B., Tehrani-Bagha, A. R., Delaine, T., Holmberg, K., Luthman, K., and Karlberg, A. T. (2014) Epoxy resin monomers with reduced skin sensitizing potency. *Chem. Res. Toxicol. 27*, 1002-1010.

(2) Gerberick, G. F., Ryan, C. A., Dearman, R. J., and Kimber, I. (2007) Local lymph node assay (LLNA) for detection of sensitization capacity of chemicals. *Methods 41*, 54-60.

(3) Basketter, D. A., Andersen, K. E., Liden, C., Van Loveren, H., Boman, A., Kimber, I., Alanko, K., and Berggren, E. (2005) Evaluation of the skin sensitizing potency of chemicals by using the existing methods and considerations of relevance for elicitation. *Contact Dermatitis 52*, 39-43.

(4) Johnson, R. S., Martin, S. A., Biemann, K., Stults, J. T., and Watson, J. T. (1987) Novel fragmentation process of peptides by collision-induced decomposition in a tandem mass spectrometer: differentiation of leucine and isoleucine. *Anal Chem 59*, 2621-2625.

(5) Biemann, K. (1988) Contributions of mass spectrometry to peptide and protein structure. *Biomed Environ Mass Spectrom 16*, 99-111.
